# Fluorogenic and Bioorthogonal Modification of RNA Using Photoclick Chemistry

**DOI:** 10.3390/biom10030480

**Published:** 2020-03-21

**Authors:** Katja Krell, Hans-Achim Wagenknecht

**Affiliations:** Institute of Organic Chemistry, Karlsruhe Institute of Technology (KIT), Fritz-Haber-Weg 6, 76131 Karlsruhe, Germany; Katja.Krell@kit.edu

**Keywords:** photochemistry, tetrazole, oligonucleotide

## Abstract

A bromoaryltetrazole-modified uridine was synthesized as a new RNA building block for bioorthogonal, light-activated and postsynthetic modification with commercially available fluorescent dyes. It allows “photoclick”-type modifications by irradiation with light (300 nm LED) at internal and terminal positions of presynthesized RNA with maleimide-conjugated fluorophores in good yields. The reaction was evidenced for three different dyes. During irradiation, the emission increases due to the formation of an intrinsically fluorescent pyrazoline moiety as photoclick product. The fluorogenecity of the photoclick reaction was significantly enhanced by energy transfer between the pyrazoline as the reaction product (poor emitter) and the photoclicked dye as the strong emitter. The RNA-dye conjugates show remarkable fluorescent properties, in particular an up to 9.4 fold increase of fluorescence, which are important for chemical biology and fluorescent imaging of RNA in cells.

## 1. Introduction

Bioorthogonal reactions are widely employed for the labeling of biomolecules, including proteins, carbohydrates and nucleic acids, with fluorescent markers and probes [1,2,3]. The widely used Cu(I)-catalyzed azide-alkyne cycloaddition (CuAAC) offers not only a broad substrate scope, but also high selectivity and fast reaction kinetics [4,5]. Although a huge variety of modifications of biomolecules by CuAAC have been reported based on Cu-chelating ligands [6], and even a few in living cells, the use of copper(I) is problematic because of its cytotoxicity, and therefore alternatives are needed [7,8,9]. Considering the reaction kinetics, both (i) the inverse electron-demand Diels-Alder reaction (iEDDA) between 1,2,4,5-tetrazines [10,11] or 1,2,4-triazines [12] and strained olefins or alkynes, and (ii) the photoclick reaction between in situ generated nitrile-imines and electron-deficient alkenes offer second order rate kinetics that are comparable to CuAAC [13,14].

**Figure 1:**
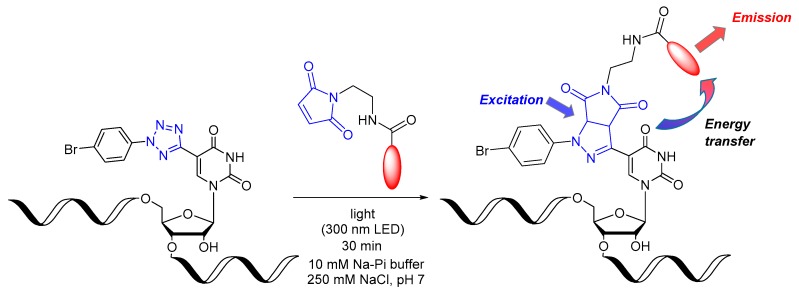
Principle of fluorogenic RNA photoclick labeling using the tetrazole-modified uridine (marked in blue, for building block structure see Scheme 1) as a photoreactive building block in single-stranded RNA and maleimide-conjugated dyes (maleimide marked in blue, dye in red). The energy transfer between the pyrazoline as a photoclick product and the attached dye yield a fluorogenic photoclick reaction.

The great advantage of the photoclick reaction compared to iEDDA reactions is the spatiotemporal control by the use of light to initiate the reaction [15,16]. An additional and important characteristic of the photoclick reaction is the intrinsic fluorogenecity of the reaction (Figure 1). Fluorogenecity is an increasingly important feature of bioorthogonal labeling strategies that allows to reduce undesired background fluorescence by using light-up probes [16]. This is particularly important if molecular imaging is performed not only with fixed cells but also with living cells, because excess dye labels cannot be simply washed away [17,18]. During irradiation, the emission increases due to the formation of an intrinsically fluorescent pyrazoline moiety as photoclick product. This work focuses on enhancing the fluorogenic properties of the photoclick reaction by energy transfer between the pyrazoline as the reaction product, which is a poor emitter, and the photoclicked dye as the strong emitter. Recently, our group reported such photoclick reactions on tetrazole-modified DNA, whereas this work focuses on the modification of RNA single strands [19]. We present herein the new phosphoramidite **9** as a synthetic RNA building block, its incorporation in two RNA sequences by automated solid-phase chemistry, and its photochemical evaluation for fluorogenic modification at internal and terminal positions in single-stranded RNA using three different commercially available dyes.

## 2. Materials and Methods

### General

All Reagents were purchased from commercial sources and used without further purification. The compounds were purified via silica gel column chromatography (230–400 mesh). The completion of the reactions was monitored via t.l.c. on silica gel F_254-_coated plates and under a 254 nm lamp. Nucleosides were stained with 3% H_2_SO_4_ in methanol followed by heating with a heat gun. ^1^H and ^13^C-NMR spectra were recorded either on a Bruker Ascend 500 MHz or Bruker Ascend 400 MHz spectrometer in DMSO-d6. Chemical shifts *δ* are given in ppm relative to tetramethylsilane, the spectra were referenced to the solvent peak (DMSO-d6, 2.50 ppm). Coupling constants *J* are given in Hz. For multiplicities, the following abbreviations are used: s (singlet), d (doublet), dd (doublet of doublets), ddd (doublet of doublets of doublets), t (triplet), dt (doublet of triplets), q (quartet), m (multiplet).

Oligonucleotide synthesis was performed on a *H-6* synthesizer by K&A Laborgeräte (Schaafheim, Germany), the coupling time of the artificial building block was increased to 24 min. After cleavage, the oligonucleotides were purified on a semi-preparative reversed-phase HPLC system (RP-C18 column, A = NH_4_HCO_3_ buffer, B = acetonitrile). The purified oligonucleotide strands were quantified photometrically using a NanoDrop ND-1000 spectrometer and the extinction coefficient of the strands (ε_260_ = 165 807 L mol^−1^ cm^−1^). Photoclicked oligonucleotides were purified via illustra^TM^ NAP-5 columns (GE Healthcare, Chicago, USA) to remove excess dyes. MS were measured on a Shimadzu Axima Confidence using 3-hydroxypicolinic acid as matrix substance. Irradiation experiments were performed in 10 mm quartz glass cuvettes and irradiated with 300 nm LEDs for defined time spans in 10 mM Na-Pi buffer, 250 mM NaCl with 2.5 μM RNA and 3.75 μM dye-maleimide conjugates at 20 °C. Spectroscopic measurements were carried out on a Cary 100 Scan UV/vis spectrometer by Varian and a Fluoromax-4 spectrofluorometer by Horiba Jobin-Yvon (Kyoto, Japan).

Synthesis of compound 2

To a stirred solution of 5-hydroxyuracil [20] (1.41 g, 9.91 mmol; 1.00 equiv.) and β-D-ribofuranose 1-acetate 2,3,5-tribenzoate (5.00 g, 9.91 mmol, 1.00 eq) in anhydrous acetonitrile (50 mL), N,O-bistrimethylsilyl acetamide (6.05 g, 29.7 mmol; 3.00 equiv.) was added. The solution was stirred for 30 min at 60 °C. Trimethylsilyl trifluoromethanesulfonate (7.71 g, 34.7 mmol; 3.50 equiv.) was added and the reaction mixture was stirred for 2 h at 60 °C. After dilution with ethyl acetate (200 mL), the solution was washed with water (3 × 300 mL) and brine (100 mL). The organic phase was dried over sodium sulfate and concentrated under vacuum. After flash chromatography (dichloromethane/methanol 40:1 – 20:1), the product was obtained as a white foam (4.07 g; 70%). Rf (dichloromethane/methanol 19:1) = 0.26. ^1^H-NMR (400 MHz, DMSO, Figure S1) *δ* = 11.55 (s, 1H), 8.02 (dd, *J* = 8.2, 1.4 Hz, 2H), 7.93 – 7.85 (m, 4H), 7.74 (t, *J* = 1.2 Hz, 1H), 7.69 – 7.61 (m, 3H), 7.51 (t, *J* = 7.8 Hz, 2H), 7.44 (td, *J* = 7.7, 3.7 Hz, 4H), 6.28 – 6.22 (m, 1H), 5.95 – 5.90 (m, 2H), 5.03 (t, *J* = 5.4 Hz, 1H), 4.75 (dt, *J* = 5.5, 2.7 Hz, 1H), 4.67 (t, *J* = 4.1 Hz, 2H), 4.19 – 4.09 (m, 2H). ^13^C-NMR (101 MHz, DMSO, Figure S2) *δ* = 165.5, 164.7, 164.6, 162.6, 150.3, 138.2, 133.9, 133.8, 133.5, 129.3, 129.3, 129.2, 128.8, 128.7, 128.6, 128.5, 114.8, 89.5, 78.7, 73.1, 70.5, 63.7, 55.9. HRMS (FAB, Figures S3 and S4): Calcd. for C_31_H_27_O_10_N_2_ [MH]+: 587.1666, found: 587.1667.

Synthesis of compound 3

**2** (2.33 g, 3.97 mmol; 1.00 equiv.) was dissolved in dichloromethane (150 mL). Dess-Martin Periodinane (15% *w/w* in dichloromethane, 9.90 mL, 13.5 g, 4.77 mmol, 1.20 equiv.) was added. The reaction mixture was stirred at r.t. for 2 h and filtered. The solvent was evaporated under reduced pressure and the crude product purified via flash chromatography (dichloromethane/methanol 60:1). The product was obtained as a white foam (1.68 g, 72%) Rf (dichloromethane/methanol 19:1) = 0.37. ^1^H-NMR (400 MHz, DMSO, Figure S5) *δ* = 11.98 (s, 1H), 9.75 (s, 1H), 8.59 (s, 1H), 8.04 – 7.97 (m, 2H), 7.89 (ddd, *J* = 16.4, 8.3, 1.4 Hz, 4H), 7.68 – 7.60 (m, 3H), 7.51 – 7.40 (m, 6H), 6.29 (d, *J* = 3.5 Hz, 1H), 6.03 (dd, *J* = 6.3, 3.5 Hz, 1H), 5.97 (t, *J* = 6.5 Hz, 1H), 4.80 (ddd, *J* = 6.5, 5.1, 3.5 Hz, 1H), 4.73 – 4.63 (m, 2H). ^13^C-NMR (101 MHz, DMSO, Figure S6) *δ* = 186.2, 165.5, 164.6, 164.5, 161.7, 149.4, 148.5, 133.9, 133.8, 133.5, 129.4, 129.3, 129.1, 128.8, 128.7, 128.6, 128.5, 111.1, 90.9, 79.3, 73.8, 70.1, 63.4. HRMS (FAB, Figures S7 and S8): Calcd. for C_31_H_25_O_10_N_2_ [MH]+: 585.1509, found: 585.1508.

Synthesis of compound 4

**3** (1.68 g, 2.87 mmol; 1.00 equiv.) and benzenesulfonyl hydrazide (494 mg, 2.87 mmol; 1.00 equiv.) were dissolved in dichloromethane (100 mL) and stirred for 2 h at 40 °C. The reaction mixture was concentrated under reduced pressure and purified via column chromatography (dichloromethane/methanol 40:1). The product was obtained as a white foam (1.74g, 82%). Rf (dichloromethane/methanol 19:1) = 0.28. ^1^H-NMR (400 MHz, DMSO, Figure S9) *δ* = 11.88 (s, 1H), 11.45 (s, 1H), 8.21 (s, 1H), 8.00 – 7.96 (m, 2H), 7.93 – 7.86 (m, 6H), 7.81 (s, 1H), 7.67 – 7.54 (m, 6H), 7.50 – 7.40 (m, 6H), 6.28 (d, *J* = 3.2 Hz, 1H), 6.03 (dd, *J* = 6.4, 3.2 Hz, 1H), 5.97 (t, *J* = 6.6 Hz, 1H), 4.76 (td, *J* = 6.0, 3.7 Hz, 1H), 4.67 (qd, *J* = 12.0, 4.7 Hz, 2H). ^13^C-NMR (101 MHz, DMSO, Figure S10) *δ* = 165.5, 164.7, 164.6, 161.9, 149.5, 140.3, 140.0, 139.0, 138.2, 133.9, 133.8, 133.5, 133.0, 132.6, 129.4, 129.3, 129.2, 129.2, 129.0, 128.7, 128.7, 128.6, 128.6, 127.6, 127.2, 107.9, 92.0, 79.0, 73.6, 70.5, 63.7. HR-MS (FAB, Figures S11 and S12) Calcd. for C_37_H_31_O_11_N_4_S_1_ [MH]+: 739.1710, found: 739.1709.

Synthesis of compound 5

**4** (890 mg 1.20 mmol; 1.00 equiv.) was dissolved in pyridine (50 mL) and cooled to -15 °C. 4-Bromobenzenediazonium tetrafluoroborate (489 mg, 1.81 mmol; 1.50 equiv.) was added and the reaction mixture was stirred for 30 min. It was diluted with dichloromethane (100 mL) and washed 3 times with 1 M hydrochloric acid, 2 times with water and once with brine. After the organic phase was dried over sodium sulfate and evaporated under reduced pressure, the crude product was redissolved in dichloromethane (5 mL) and precipitated by addition of *n*-hexane (200 mL). The precipitation was repeated, until no further precipitation occurred. The product was obtained as a pale orange powder (451 mg, 48%). Rf (dichloromethane/methanol 19:1) = 0.51. ^1^H-NMR (400 MHz, DMSO, Figure S13) *δ* = 12.01 (s, 1H), 8.68 (s, 1H), 8.00 – 7.91 (m, 6H), 7.90 – 7.86 (m, 4H), 7.65 (dt, *J* = 11.1, 7.5 Hz, 2H), 7.52 – 7.41 (m, 5H), 7.34 (t, *J* = 7.7 Hz, 2H), 6.36 (d, *J* = 3.8 Hz, 1H), 6.06 (dd, *J* = 6.2, 3.8 Hz, 1H), 6.02 (t, *J* = 6.2 Hz, 1H), 4.84 (dt, *J* = 6.3, 3.9 Hz, 1H), 4.74 (dd, *J* = 12.3, 3.3 Hz, 1H), 4.65 (dd, *J* = 12.3, 4.6 Hz, 1H). ^13^C-NMR (101 MHz, DMSO, Figure S14) *δ* = 165.5, 164.6, 164.6, 159.8, 159.5, 149.6, 143.3, 135.2, 133.9, 133.8, 133.3, 133.1, 129.4, 129.3, 129.2, 129.0, 128.8, 128.7, 128.6, 128.5, 122.9, 121.7, 102.1, 89.9, 79.4, 73.9, 70.3, 63.6. HRMS (FAB, Figures S15 and S16): Calcd. for C_37_H_28_O_9_N_6_Br [MH]+: 779.1101, found: 779.1102.

Synthesis of compound 6

In a pressure tube, **5** (385 mg, 0.494 mmol; 1.00 equiv.) was suspended in 7M ammonia in methanol (50 mL) and stirred at 80 °C for 24 h. The solvent was removed under reduced pressure and the crude product was purified via flash chromatography (dichloromethane/methanol 10:1). The product was obtained as a white powder (150 mg; 65%). Rf (dichloromethane/methanol 9:1) = 0.18. ^1^H-NMR (500 MHz, DMSO, Figure S17) *δ* = 11.83 (s, 1H), 8.88 (s, 1H), 8.08 – 8.04 (m, 2H), 7.91 – 7.87 (m, 2H), 5.86 (d, J = 4.5 Hz, 1H), 5.51 (d, J = 5.3 Hz, 1H), 5.18 (t, J = 4.7 Hz, 1H), 5.13 (d, J = 5.4 Hz, 1H), 4.14 (q, J = 4.9 Hz, 1H), 4.03 (q, J = 5.0 Hz, 1H), 3.92 (dt, J = 5.0, 2.6 Hz, 1H), 3.69 (ddd, J = 11.9, 4.7, 2.8 Hz, 1H), 3.59 (ddd, J = 12.0, 4.6, 2.7 Hz, 1H). ^13^C-NMR (126 MHz, DMSO, Figure S18) δ = 160.0, 159.9, 150.0, 142.6, 135.3, 133.1, 123.0, 121.8, 101.7, 88.7, 84.8, 74.2, 69.5, 60.3. HRMS (FAB Figures S19 and S20): Calcd. for C_16_H_16_O_6_N_6_Br [MH]+: 467.0315, found: 467.0314.

Synthesis of compound 7

**6** (315 mg, 0.674 mmol; 1.00 equiv.) was dissolved in anhydrous DMF (5 mL). 4,4′-Dimethoxytrityl chloride (228 mg, 0.674 mmol; 1.00 equiv.) and silver(II) nitrate (114 mg, 0.674 mmol, 1.00 equiv.) were added and the reaction mixture was stirred for 2 h at r.t. The solvent was evaporated under reduced pressure and the crude product purified via column chromatography (dichloromethane/methanol 50:1 → 20:1 + 0.1 % triethylamine). The pure product was obtained as a white powder (315 mg; 61%). Rf (dichloromethane/methanol 20:1) = 0.22. ^1^H-NMR (500 MHz, DMSO, Figure S20) δ = 11.87 (s, 1H), 8.44 (s, 1H), 7.73 – 7.68 (m, 2H), 7.67 – 7.63 (m, 2H), 7.33 (dd, J = 8.5, 1.3 Hz, 2H), 7.25 – 7.18 (m, 4H), 7.15 (t, J = 7.8 Hz, 2H), 7.07 – 7.02 (m, 1H), 6.71 (d, J = 8.9 Hz, 4H), 5.82 (d, J = 3.4 Hz, 1H), 5.65 (d, J = 5.0 Hz, 1H), 5.16 (d, J = 6.3 Hz, 1H), 4.24 (td, J = 5.0, 3.4 Hz, 1H), 4.18 – 4.12 (m, 1H), 4.06 (dt, J = 6.2, 2.9 Hz, 1H), 3.63 (d, J = 2.0 Hz, 6H), 3.23 (d, J = 3.3 Hz, 2H). ^13^C-NMR (126 MHz, DMSO, Figure S21) δ 160.1, 159.4, 157.8, 149.8, 149.6, 144.4, 141.3, 135.5, 135.3, 135.0, 132.7, 129.5, 129.5, 127.6, 127.6, 126.5, 123.9, 122.5, 121.5, 112.9, 112.9, 101.7, 89.7, 85.7, 82.6, 74.1, 69.2, 62.5, 54.8, 54.8. MS (MALDI-TOF, Figure S22): Calcd. for C_37_H_32_BrN_6_NaO_8_ [M+Na]^+^: 790.1363, found: 789.43.

Synthesis of compound 8

In a brown glass flask, **7** (500 mg, 0.649 mmol; 1.00 equiv.) was dissolved in anhydrous tetrahydrofuran (5 mL). Silver (II) nitrate (143 mg, 0.844 mmol; 1.30 equiv.) and anhydrous pyridine (209 μL, 206 mg; 2.60 mmol; 4.00 equiv.) were added and stirred for 20 min under exclusion of light. *tert*-Butyldimethylsilyl chloride (137 mg, 0.909 mmol; 1.40 equiv.) were added and the reaction mixture was stirred at r. t. for 6 h. The solution was diluted with ethyl acetate (20 mL) and filtrated. The solvent was removed under reduced pressure and the crude product was purified via column chromatography (dichloromethane/methanol 50:1 → 30:1 + 0.1 % triethylamine). The product was obtained as a white powder (125 mg; 36%). Rf (dichloromethane/methanol 20:1) = 0.27. ^1^H-NMR (500 MHz, DMSO, Figure S24) *δ* = 11.90 (s, 1H), 8.48 (s, 1H), 7.71 – 7.67 (m, 2H), 7.65 – 7.60 (m, 2H), 7.36 – 7.31 (m, 2H), 7.21 (dd, *J* = 10.8, 8.9 Hz, 4H), 7.13 (t, *J* = 7.7 Hz, 2H), 7.07 – 7.02 (m, 1H), 6.71 (d, *J* = 2.5 Hz, 2H), 6.69 (d, *J* = 2.6 Hz, 2H), 5.80 (d, *J* = 3.1 Hz, 1H), 5.20 (d, *J* = 6.0 Hz, 1H), 4.37 (dd, *J* = 4.5, 3.1 Hz, 1H), 4.18 – 4.12 (m, 1H), 4.10 (dt, *J* = 6.2, 2.6 Hz, 1H), 3.63 (s, 3H), 3.62 (s, 3H), 3.25 (s, 2H), 0.89 (s, 9H), 0.13 (s, 3H), 0.10 (s, 3H). ^13^C-NMR (126 MHz, DMSO, Figure S25) *δ* = 160.0, 159.3, 157.8, 157.8, 149.7, 144.4, 140.9, 135.4, 135.2, 135.0, 132.6, 129.5, 129.5, 127.6, 127.5, 126.5, 122.5, 121.5, 112.9, 112.9, 101.7, 89.7, 85.8, 82.5, 76.3, 69.0, 62.2, 54.8, 54.8, 25.7, 18.0, -3.2, -4.7, -5.0. HRMS (FAB, Figures S26 and S27): Calcd. for C_43_H_47_O_8_N_6_BrSi [M]^+^: 882.2408, found: 882.2411.

Synthesis of compound 9

**8** (120 mg 0.135 mmol: 1.00 equiv.) was lyophilized out of benzene, then dissolved in anhydrous dichloromethane (4 mL). Diisopropylethyl amine (71 μL, 0.407 mmol; 3.00 equiv.) and 2-cyanoethyl-N,N-diisopropylchlorophoshoramidite (45 μL, 0.204 mmol; 1.50 eq) were added and stirred for 6 h at r.t.. The crude product was purified via column chromatography (dichloromethane/acetone 5:1 + 0.1% triethylamine). The product was obtained as a white powder (131 mg; 87%). Rf (dichloromethane/acetone 5:1) = 0.28. ^31^P-NMR (202 MHz, DMSO, Figure S28) *δ* = 148.89, 148.74. MS (MALDI-TOF, Figure S29): Calcd. for C_52_H_64_O_9_N_8_BrP [M+Na]^+^: 1105.39, found: 1105.10.

## 3. Results and Discussion

**Scheme 1:**
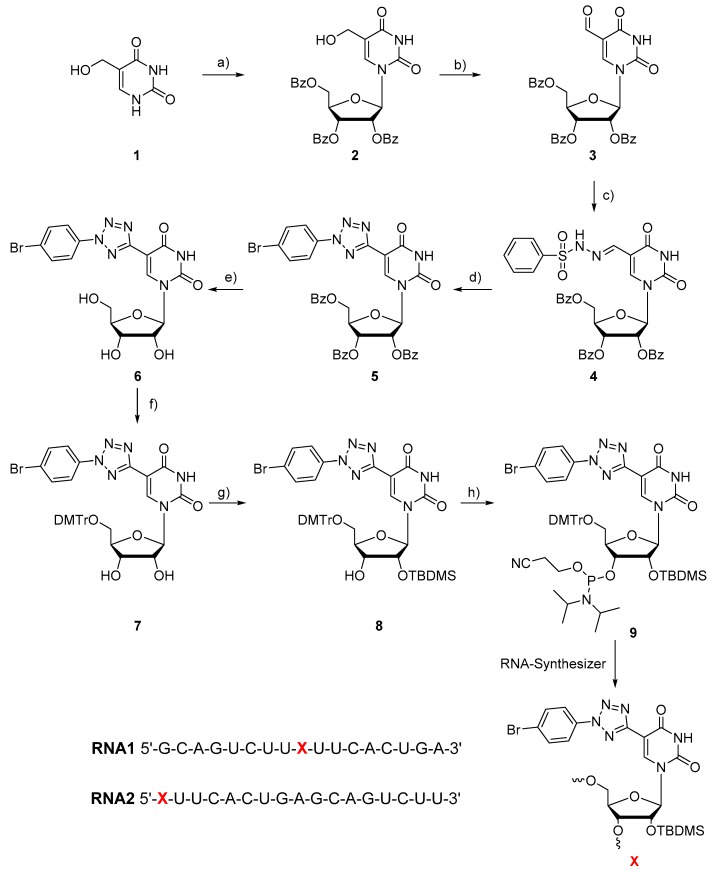
Synthesis of RNA building block **9**: （**a**) 2,3,5-Tribenzoyl-β-D-ribofuranoside-1-acetate, N,O-bis(trimethylsilyl)acetamide, trimethylsilyl trifluoromethanesulfonate, acetonitrile, 60 °C, 2 h, 70%. （**b**) Dess-Martin periodinane, CH_2_Cl_2_, r.t., 2 h, 72%. （**c**) Benzenesulfonyl hydrazide, dichloromethane, 40 °C, 2 h, 82%. （**d**) 4-bromobenzenediazonium tetrafluoroborate, pyridine, −15 °C, 0.5 h, 48%. （**e**) Ammonia (7M) in MeOH, 80 °C, 24 h, 65%. （**f**) DMT-Cl, silver(II) nitrate, pyridine, r.t., 2 h, 61%. （**g**) AgNO_3_, TBDMS-Cl, pyridine, r.t., 6 h, 36%. （**h**) DIPEA, 2-cyanoethyl *N,N*-diisopropyl-chlorophosphoramidite, CH_2_Cl_2_, r. t., 6 h, 87%.

In principle, diaryltetrazoles are chemically stable but they are also bioorthogonally reactive groups when excited by UV-B light [21]. The RNA building block **9** bears the reactive tetrazole at its position 5. In order to keep the modified nucleotide as small as possible the uracil moiety replaces one of the aryl groups of conventional diaryltetrazoles. The bromo substituent at the opposite aryl group is needed for efficient photoclick chemistry [19] presumably by its heavy-atom effect on populating the photochemically active triplet state [22]. The synthetic route (Scheme 1) was adapted from the synthesis of a similar 2′-deoxyuridine building block for DNA recently published by our group [19] but contains improved synthetic procedures and a different protection scheme due to the presence of the 2′-hydroxy group. Accordingly, the 2′,3′,5′-tribenzoyl-protected 5-hydroxymethyluridine (**2**) was synthesized by coupling 5-hydroxymethyluracil that was prepared following published procedures [20] with the commercially available 1-acetate-2,3,5-tribenzoate of β-D-ribofuranoside using modified Vorbrüggen conditions in 70% yield [23]. The 5-hydroxymethyl group was oxidized by Dess-Martin periodinane in 72% yield [24]. The conversion of the aldehyde moiety of **3** to the hydrazone **4** was achieved in 82% yield by treatment with benzenesulfonyl hydrazide [25]. Upon addition of the commercially available 4-bromobenzenediazonium tetrafluoroborate, the tetrazole **5** was formed in 48% yield [25]. The next steps comprised the deprotection of all benzoyl groups of **5** by 7 M ammonia in methanol to obtain nucleoside **6**, selective protection of the 5′-hydroxy group of **6** by the dimethoxytrityl group to get nucleoside **7**, and protection of the 2′ group of **7** with a *tert*-butyldimethyl silyl group. The latter reaction gave nucleoside **8** in 36% yield using silver nitrate as catalyst [26]. Finally, the hydroxy group at the 3′-position of **8** was phosphitylated by 2-cyanoethyl *N,N*-diisopropylchlorophosphoramidite to yield the final RNA phosphoramidite **9**. The RNA building block **9** was then incorporated via solid-phase synthesis into two different RNA sequences with the tetrazole moiety either at an internal (**RNA1**) or at a 5′-terminal position (**RNA2**) of the sequences. The synthesized oligonucleotide strands were purified by semi-preparative HPLC, identified by MALDI-TOF mass spectrometry and quantified by their UV/vis absorbance at 260 nm.

**Figure 2:**
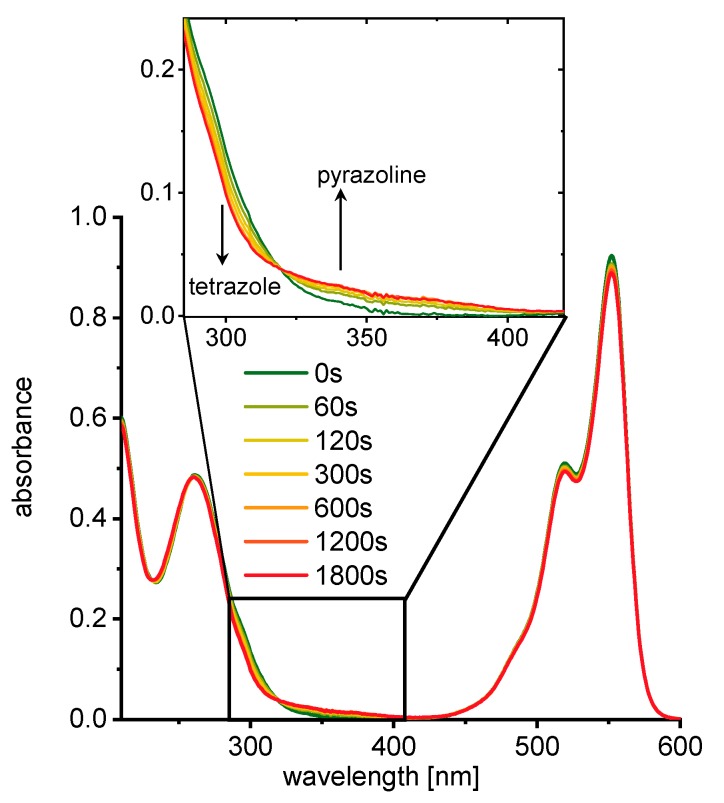
UV/Vis absorbances recorded during irradiation of **RNA1** (2.5 μM) with 1.50 equiv. AF555-maleimide (3.75 μM) in 10 mM Na-P_i_ buffer (250 mM NaCl, pH 7).

As reaction partners for the photoclick labeling of **RNA1** and **RNA2**, three commercially available maleimide-dye conjugates were chosen, including sulfo-Cy3 (Cy3), AlexaFluor555 (AF555) and AlexaFluor647 (AF647). During the 30 min reaction course, the UV/Vis absorbance and fluorescence changes were recorded (representatively shown for **RNA1** and the AF555 modification in Figure 2, for the others see Figures S31–S41). The tetrazole absorbance is visible as tailing side band at 300 nm, which is at the border of the strong RNA absorbance. According to these measured UV/Vis spectra, 300 nm light from the appropriate LED was chosen to induce the photoclick reaction. The RNA building block **6** has an extinction coefficient of ε_300_=20.300 M^−1^cm^−1^ which is significantly larger compared to the extinction coefficients of the natural RNA components A, C, G and U (ε_300_=60–260 M^−1^ cm^−1^, see Supporting Information). Accordingly, in **RNA1** and **RNA2** the excitation selectivity for the tetrazole at 300 nm is approximately 10:1 that excludes an inner filter effect by the RNA. In the presence of 1.5 equiv. of the dye-maleimide conjugate, a new and broad band between 320 and 420 nm is formed, which can be assigned to the pyrazoline moiety formed in the RNA product, whereas the tetrazole absorbance between 280 and 320 nm concomitantly decreases. The UV/Vis spectra show distinct isosbestic points that indicate the clean conversions from the tetrazoles to the pyrazolines in all cases without the formation of a long-living intermediate. According to these UV/Vis absorbance changes, the reaction is finished after 30 min irradiation.

The oligonucleotide products were evidenced by MALDI-TOF mass spectrometric analysis (Figures S42–S53). After chromatographic removal of excess and unreacted dyes (see SI for experimental details and Figures S54–S57) the sample concentrations were calculated based on their UV/Vis absorbances. The yields of the postsynthetic photoclick modifications were determined for each sample separately after the purification (The Cy3-conjugates show the lowest yields of 27% for **RNA1** and 31% for **RNA2,** albeit its strong increase in fluorescence (*vide infra*). Exemplarily, the photoclick conjugation was repeated with 10.0 equiv. of the Cy3-maleimide and **RNA2** to determine whether the yield could be further enhanced. In fact, the yield of this irradiation reaction was 70%, which of course caused a remarkably higher fluorescence. With respect to potential cell experiments, however, such an excess of dye might be critical due to the high background fluorescence of the dye. The highest yields of 78% for **RNA1** and 84% for **RNA2** could be achieved by reaction with the AF555 maleimide (1.50 equiv.), although these samples display the lowest absolute fluorescence (see Figures S38 and S40 and Table 1). The Cy3-conjugates show the lowest yields of 27% for **RNA1** and 31% for **RNA2**, albeit its strong increase in fluorescence (*vide infra*). Exemplarily, the photoclick conjugation was repeated with 10.0 equiv. of the Cy3-maleimide and **RNA2** to determine whether the yield could be further enhanced. In fact, the yield of this irradiation reaction was 70%, which of course caused a remarkably higher fluorescence. With respect to potential cell experiments, however, such an excess of dye might be critical due to the high background fluorescence of the dye. The highest yields of 78% for **RNA1** and 84% for **RNA2** could be achieved by reaction with the AF555 maleimide (1.50 equiv.), although these samples display the lowest absolute fluorescence (see Figures S38 and S40).

**Figure 3:**
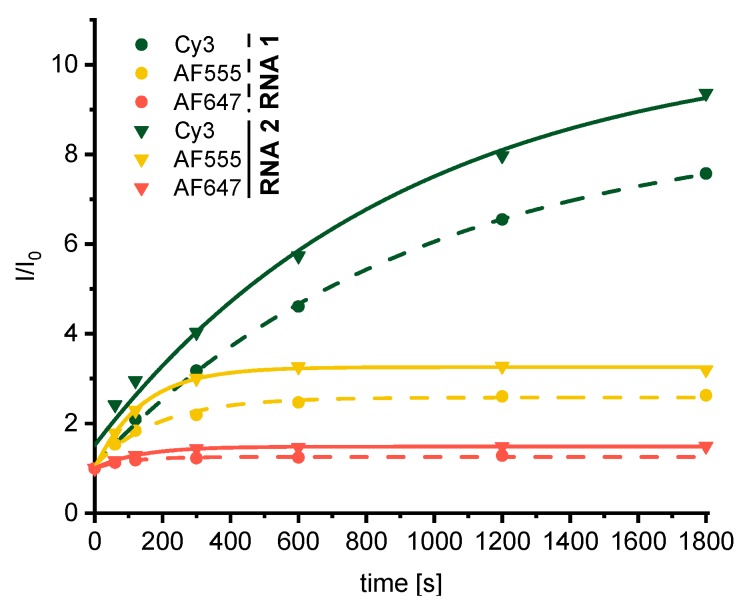
Fluorescence intensity changes during the photoclick modification of **RNA1** (2.5 μM, dashed lines) and **RNA2** (2.5 μM, solid lines) in the presence of Cy3-maleimide, AF555-maleimide and AF647 maleimide (each 3.75 μM, 1.5 equiv.); irradiation at 300 nm, fluorescence excitation at 358 nm, in 10 mM Na-P_i_ buffer (250 mM NaCl, pH 7). I_0_ is the fluorescence intensity before the irradiation.

The excitation of the fluorescence was set to 358 nm, which is close to the absorption maximum of the pyrazoline moiety. The extinction of the applied dyes Cy3, AF647 and AF555 is very low at 358 nm, which nearly completely eliminates direct excitation of these dyes. Thus, the observed fluorescence can be assigned to the pyrazoline as direct photoclick product and the attached dyes that were coupled to the RNA and light up as a result of the energy transfer from the pyrazoline chromophore to these dyes. The recorded fluorescence spectra during the irradiations (Figure 3) show only minor increases of fluorescence intensity between 400 and 500 nm, which is the characteristic emission range of the pyrazoline chromophore, but strong increases of fluorescence intensity in the emission range of the attached dyes (with maxima at 564 nm for Cy3, 666 nm for AF647 and 565 nm for AF555). Based on the assumption that direct excitation of these dyes can be excluded, as mentioned above, this result clearly evidences the energy transfer between the pyrazoline and the dyes [19]. However, the gain of fluorescence intensity strongly depends on both the type of fluorophore (Cy3, AF647, AF655) and the position of the photoclick modification in the RNA strand (terminal, internal). In general, the terminally modified **RNA2** displays a higher fluorogenicity than the internally modified sequence **RNA1**. In case of the Cy3 dye, remarkable 7.5-fold (**RNA1**) and 9.4-fold (**RNA2**) fluorescence intensity increases could be achieved. Even though Cy3 and AF555 are spectroscopically similar, the energy transfer between the pyrazoline moiety and Cy3 seems to be more efficient compared to AF555 because only 2.6-fold (**RNA1**) and 3.2-fold (**RNA2**) fluorescence intensity increases were yielded with AF555. Although structural details of both AF-maleimides are reported in literature [27], the exact structure was not provided by the manufacturer of the dyes. Therefore, the varying energy transfer efficiency cannot conclusively be explained, but we assume differing Förster radii or competing photochemical processes could be possible explanations for the lower efficiency. The lowest fluorescence gains were achieved with the AF647 dye.

## 4. Conclusions

In conclusion, we present an efficient synthetic route to the new tetrazole-modified phosphoramidite building block **9**. The uridine substitutes one of the aryl groups of the photoactive diaryltetrazole. This modification was incorporated internally and 5′-terminally into two RNA strands using conventional automated solid-phase chemistry. Photoclick reactions were carried out as postsynthetic modifications by irradiation with 300 nm light (LED) in aqueous media and in the presence of three different commercially available dye-maleimide conjugates (Cy3, AF555 and AF647). The yields vary significantly for the different dye-maleimide conjugates: AF555 maleimide produces the highest yields of up to 84% (**RNA2**), whereas the Cy3-maleimide shows smaller yields down to 27% (**RNA1**). In contrast, Cy3 provides the highest fluorogenic effect by the 7.5-fold (**RNA1**) to 9.4-fold (**RNA2**) fluorescence intensity increase, which is nearly one magnitude of order increase of fluorescence intensity. The concept has a broad substrate scope, both with respect to the dye-maleimide conjugates and with respect to the position within RNA sequence. This photoclick chemistry is a highly promising candidate for cell applications. Although the 300 nm light is in principle harmful to biological cells, the short irradiation times may be tolerable. Due to the fact that cytotoxic copper salts are avoided and the application of time allows spatiotemporal resolution, this postsynthetic RNA chemistry is a new tool for fluorescent imaging of RNA in biological cells.

## Figures and Tables

**Table 1:** Yields of the photoclick reaction. **RNA1** or **RNA2** (2.5 µM) were irradiated with 300 nm light (LED) in the presence of 1.50 equiv (3.75 µM) Cy3-maleimide, AF555-maleimide and AF647-maleimide, respectively, in 10 mM Na-P_i_ buffer (250 mM NaCl, pH 7).

Dye Adduct of	RNA1	RNA2
Cy3	27%	31% (70%) ^a^
AF555	78%	84%
AF647	48%	48%

^a^ after reaction in the presence of 10.0 equiv. Cy3-maleimide.

## Supplementary Materials

The following are available online at https://www.mdpi.com/2218-273X/10/3/480/s1. **Figure S1.** ^1^H NMR spectrum (400 MHz) of **2**. Spectrum contains traces of dichloromethane (δ = 5.76 ppm). **Figure S2.** ^13^C NMR spectrum (101 MHz) of **2**. Spectrum contains traces of dichloromethane (δ = 54.8 ppm). **Figure S3.** MS (FAB) analysis of **2**. **Figure S4.** HR-MS (FAB) analysis of **2**. **Figure S5.** ^1^H NMR spectrum (400 MHz) of **3**. Spectrum contains traces of dichloromethane (δ = 5.76 ppm). **Figure S6.** ^13^C NMR spectrum (101 MHz) of **3**. Spectrum contains traces of dichloromethane (δ = 54.8 ppm). **Figure S7.** MS (FAB) analysis of **3**. **Figure S8.** HR-MS (FAB) analysis of **3**. **Figure S9.** ^1^H NMR spectrum (400 MHz) of **4**. Spectrum contains traces of toluene (δ = 7.25 ppm, 7.18 ppm, 2.30 ppm), dichloromethane (δ = 5.76 ppm) and ethyl acetate (δ = 1.17 ppm, 4.03 ppm, 1.99 ppm). **Figure S10.** ^13^C NMR spectrum (101 MHz) of **4**. Spectrum contains traces of toluene (δ = 137.4 ppm, 128.9 ppm, 128.2 ppm, 125.3 ppm, 21.0 ppm), dichloromethane (δ = 54.9 ppm) and ethyl acetate (δ = 170.3 ppm, 59.8 ppm, 20.7 ppm, 14.1 ppm). **Figure S11.** MS (FAB) analysis of **4**. **Figure S12.** HR-MS (FAB) analysis of **4. Figure S13.** ^1^H NMR spectrum (400 MHz) of **5**. Spectrum contains traces of dichloromethane (δ = 5.76 ppm). **Figure S14.** ^13^C NMR spectrum (101 MHz) of **5**. **Figure S15.** MS (FAB) analysis of **5**. **Figure S16.** HR-MS (FAB) analysis of **5**. **Figure S17.** ^1^H NMR spectrum (500 MHz) of **6**. The spectrum contains traces of methanol (δ = 4.01 ppm, 3.16 ppm). **Figure S18.** ^13^C NMR spectrum (126 MHz) of **6**. The spectrum contains traces of methanol (δ = 48.6 ppm). **Figure S19.** MS (FAB) analysis of **6**. **Figure S20.** HR-MS (FAB) analysis of **6**. **Figure S21.** ^1^H NMR spectrum (500 MHz) of **7**. Spectrum contains traces of dichloromethane (δ = 5.76ppm). **Figure S22.** ^13^C NMR spectrum (126 MHz) of **7**. Spectrum contains traces of dichloromethane (δ = 54.9 ppm). **Figure S23.** MS (MALDI-TOF) analysis of **7**. **Figure S24.** ^1^H NMR spectrum (500 MHz) of **8**. Spectrum contains traces of dichloromethane (δ = 5.76 ppm). **Figure S25.** ^13^C NMR spectrum (126 MHz) of **8**. Spectrum contains traces of dichloromethane (δ = 54.9 ppm). **Figure S26.** MS (FAB) analysis of **8**. **Figure S27.** HR-MS (FAB) analysis of **8**. **Figure S28.** ^31^P NMR spectrum (202 MHz) of **9**. **Figure S29.** MS (MALDI-TOF) analysis of **9**. **Figure S30.** UV/Vis absorbance of **RNA1** and **RNA2** (2.5 μM) in 10 mM Na-P_i_ buffer, 250 mM NaCl, pH 7. The spectra were normalized to evaluate the relative tetrazole absorbances. **Figure S31.** UV/Vis absorbance recorded during reaction of **RNA1** (2.5 μM) with Cy3-maleimide (3.75 μM, 1.50 equiv.), irradiated at 300 nm (LED) in 10 mM Na-P_i_ buffer, 250 mM NaCl, pH 7. **Figure S32.** Fluorescence recorded during reaction of **RNA1** (2.5 μM) with Cy3-maleimide (3.75 μM, (1.50 equiv.), irradiated at 300 nm (LED) in 10 mM Na-P_i_ buffer, 250 mM NaCl, pH 7. Fluorescence excitation at 358 nm. **Figure S33.** Fluorescence recorded during reaction of **RNA1** (2.5 μM) with AF555-maleimide (3.75 μM, (1.50 equiv.), irradiated at 300 nm (LED) in 10 mM Na-P_i_ buffer, 250 mM NaCl, pH 7. Fluorescence excitation at 358 nm. **Figure S34.** UV/Vis absorbance recorded during reaction of **RNA1** (2.5 μM) with AF647-maleimide (3.75 μM, 1.50 equiv.), irradiated at 300 nm (LED) in 10 mM Na-P_i_ buffer, 250 mM NaCl, pH 7. **Figure S35.** Fluorescence recorded during reaction of **RNA1** (2.5 μM) with AF647-maleimide (3.75 μM, 1.50 eq), irradiated at 300 nm (LED) in 10 mM Na-P_i_ buffer, 250 mM NaCl, pH 7. Fluorescence excitation at 358 nm. **Figure S36.** UV/vis absorbance recorded during reaction of **RNA2** (2.5 μM) with Cy3-maleimide (3.75 μM, 1.50 equiv.), irradiated at 300 nm (LED) in 10 mM Na-P_i_ buffer, 250 mM NaCl, pH 7. **Figure S37.** Fluorescence recorded during reaction of **RNA2** (2.5 μM) with Cy3-maleimide (3.75 μM, 1.50 equiv.), irradiated at 300 nm (LED) in 10 mM Na-P_i_ buffer, 250 mM NaCl, pH 7. Fluorescence excitation at 358 nm. **Figure S38.** UV/Vis absorbance recorded during reaction of **RNA2** (2.5 μM) with AF555-maleimide (3.75 μM, 1.50 equiv.), irradiated at 300 nm (LED) in 10 mM Na-P_i_ buffer, 250 mM NaCl, pH 7. **Figure S39.** Fluorescence recorded during reaction of **RNA2** (2.5 μM) with AF555-maleimide (3.75 μM, 1.50 equiv.), irradiated at 300 nm (LED) in 10 mM Na-P_i_ buffer, 250 mM NaCl, pH 7. Fluorescence excitation at 358 nm. **Figure S40.** UV/Vis absorbance recorded during reaction of **RNA2** (2.5 μM) with AF647-maleimide (3.75 μM, 1.50 equiv.), irradiated at 300 nm (LED) in 10 mM Na-P_i_ buffer, 250 mM NaCl, pH 7. **Figure S41.** Fluorescence recorded during reaction of **RNA2** (2.5 μM) with AF647-maleimide (3.75 μM, 1.50 equiv.), irradiated at 300 nm (LED) in 10 mM Na-P_i_ buffer, 250 mM NaCl at pH 7. Fluorescence excitation at 358 nm. **Figure S42.** MS (MALDI-TOF) analysis of **RNA1**. Calculated mass [M^+^]: 5544.6; m/z = 5546.99 [M^+^], 5584.98 [M+K^+^]. **Figure S43.** MS (MALDI-TOF) analysis of **RNA1**-Cy3 adduct. Calculated mass [M^+^]: 6253.9; m/z = 5523.65 [RNA1-N_2_^+^], 5541.82 [RNA1-N_2_+H_2_O^+^], 6261.44 [M^+^]. **Figure S44.** MS (MALDI-TOF) analysis of **RNA1**-AF555 adduct. Calculated mass: 6485.9 [M^+^]; m/z = 5518.14 [RNA1-N_2_^+^], 5536.19 [RNA1-N_2_+H_2_O^+^], 6486.22 [M^+^]. The molecular mass of AF555-maleimide was reported in literature and verified by MS (MALDI-TOF) analysis. **Figure S45.** Zoomed area of MS (MALDI-TOF) analysis (**Figure S44**) of **RNA1**-AF555 adduct. **Figure S46.** MS (MALDI-TOF) analysis of **RNA1**-AF647 adduct. Calculated Mass [M^+^]: 6497.9; m/z = 5519.49 [RNA1-N_2_^+^], 5537.50 [RNA1-N_2_+H_2_0^+^], 6499.90 [M^+^]. The molecular mass of AF647-maleimide was reported in literature and verified by MS (MALDI-TOF) analysis. **Figure S47.** Zoomed area of MS (MALDI-TOF) analysis (**Figure S46**) of **RNA1**-AF647 adduct. **Figure S48.** MS (MALDI-TOF) analysis of **RNA2**. Calculated mass [M^+^]: 5544.6; m/z = 5546.50 [M^+^], 5584.48 [M+K^+^],5622.49 [M+2K^+^], 5659.92 [M+3K^+^]. **Figure S49.** MS (MALDI-TOF) analysis of **RNA2**-Cy3 adduct. Calculated mass [M^+^]: 6253.9; m/z = 5516.65 [RNA2-N_2_^+^], 5534.67 [RNA2-N_2_+H_2_O^+^], 6254.67 [M^+^]. **Figure S50.** MS (MALDI-TOF) analysis of **RNA2**-AF555 adduct. Calculated Mass [M^+^]: 6485.9; m/z = 5519.45 [RNA2-N_2_^+^], 5537.50 [RNA2-N_2_+H_2_O^+^], 6485.06 [M^+^]. The molecular mass AF555-maleimide was reported in literature and verified by MS (MALDI-TOF) analysis. **Figure S51.** Zoomed area of MS (MALDI-TOF) analysis (**Figure S50**) of **RNA2**-AF555 adduct. **Figure S52.** MS (MALDI-TOF) analysis of **RNA2**-AF647 adduct. Calculated mass [M^+^]: 6497.9; m/z = 5519.01 [RNA2-N_2_^+^], 5537.02 [RNA2-N_2_+H_2_O^+^], 6499.74 [M^+^]. The molecular mass AF647-maleimide was reported in literature and verified by MS (MALDI-TOF) analysis. **Figure S53.** Zoomed area of MS (MALDI-TOF) analysis (**Figure S53**) of **RNA2**-AF647 adduct. **Figure S54.** UV/vis absorbance of “photoclicked“ **RNA1** dye adducts (reaction with 1.50 equiv. dye-maleimide) strands after purification. c_AF647_ = 1.19 µM ≙ 48% yield, c_AF555_ = 1.96 µM ≙ 78% yield, c_Cy3_ = 0.67 µM ≙ 27% yield. **Figure S55.** Fluorescence of “photoclicked” **RNA1** dye adducts (reaction with 1.50 equiv. dye-maleimide) after purification. c_AF647_ = 1.19 µM, c_AF555_ = 1.96 µM, c_Cy3_ = 0.67 µM. **Figure S56.** UV/vis absorbance of “photoclicked“ **RNA2** dye adducts (reaction with 1.50 equiv. dye-maleimide) after purification. c_AF647_ = 1.20 µM ≙ 48% yield, c_AF555_ = 2.10 µM ≙ 84% yield, c_Cy3_ = 0.77 µM ≙ 31% yield. **Figure S57.** Fluorescence of “photoclicked” **RNA2** dye adducts (after reaction with 1.50 equiv. dye-maleimide) after purification. c_AF647_ = 1.20 µM, c_AF555_ = 2.10 µM, c_Cy3_ = 0.77 µM. **Figure S58.** UV/vis absorbance of “photoclicked“ **RNA1**-Cy3 adduct (reaction with 10.0 equiv. Cy3-maleimide) after purification. c_Cy3_ = 1.76 µM ≙ 70% yield. **Figure S59.** Fluorescence spectrum of “photoclicked” **RNA2**-Cy3 adduct (reaction with 10.0 equiv. Cy3-maleimide) after purification. c_Cy3_ = 1.76 µM. **Figure S60.** UV/vis absorbance of A, C, G, U and **6** in comparison. **Table S1.** Molar extinction coefficients of the natural bases and the artificial nucleoside **6**.
