# Supplementary material for: Fluorogenic and Bioorthogonal Modification of RNA Using Photoclick Chemistry
Source: Biomolecules. 2020 Mar 21;10(3):480. doi: 10.3390/biom10030480 (PMC7175119; doi:10.3390/biom10030480)
Supplement: Supplementary file 1 [file biomolecules-10-00480-s001.pdf]

biomolecules

MDPI

Fluorogenic and bioorthogonal modification of RNA using photoclick chemistry

Katja Krell and Hans-Achim Wagenknecht

Supplementary Information

Institute of Organic Chemistry

Karlsruhe Institute of Technology (KIT)

Fritz-Haber-Weg 6

76131 Karlsruhe, Germany

E-Mail: Wagenknecht@kit.edu

Table of Contents

1. $^{1}H/^{13}C$ NMR spectra and MS analyses 2

2. Optical Spectroscopy 25

3. MALDI spectra of RNA strands 31

4. Determination of yields 40

5. Calculation of extinction coefficients 44

6. References 44

biomolecules

MDPI

1. ^{1}H/^{13}C NMR spectra and MS analyses

Compound 2

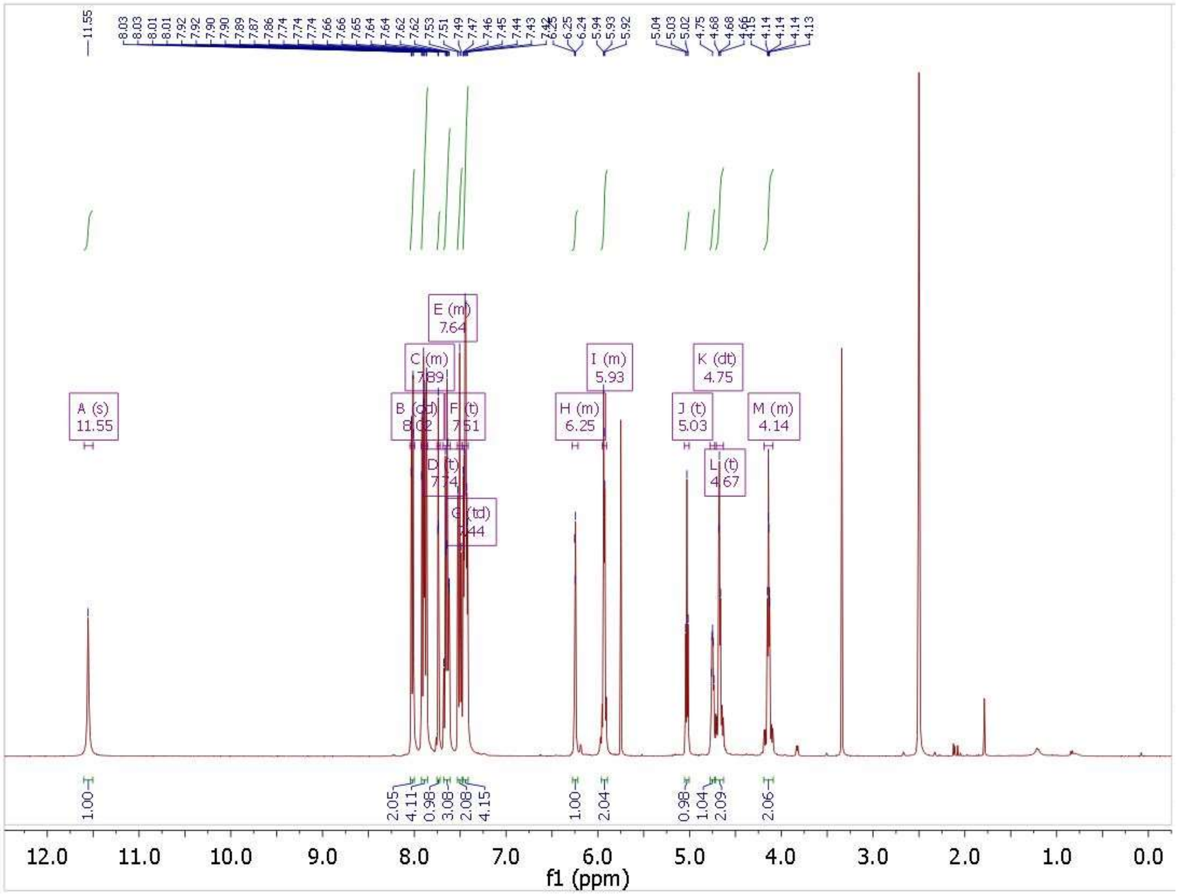

Figure S1. ^{1}H NMR spectrum (400 MHz) of **2**. Spectrum contains traces of dichloromethane (δ=5.76 ppm).

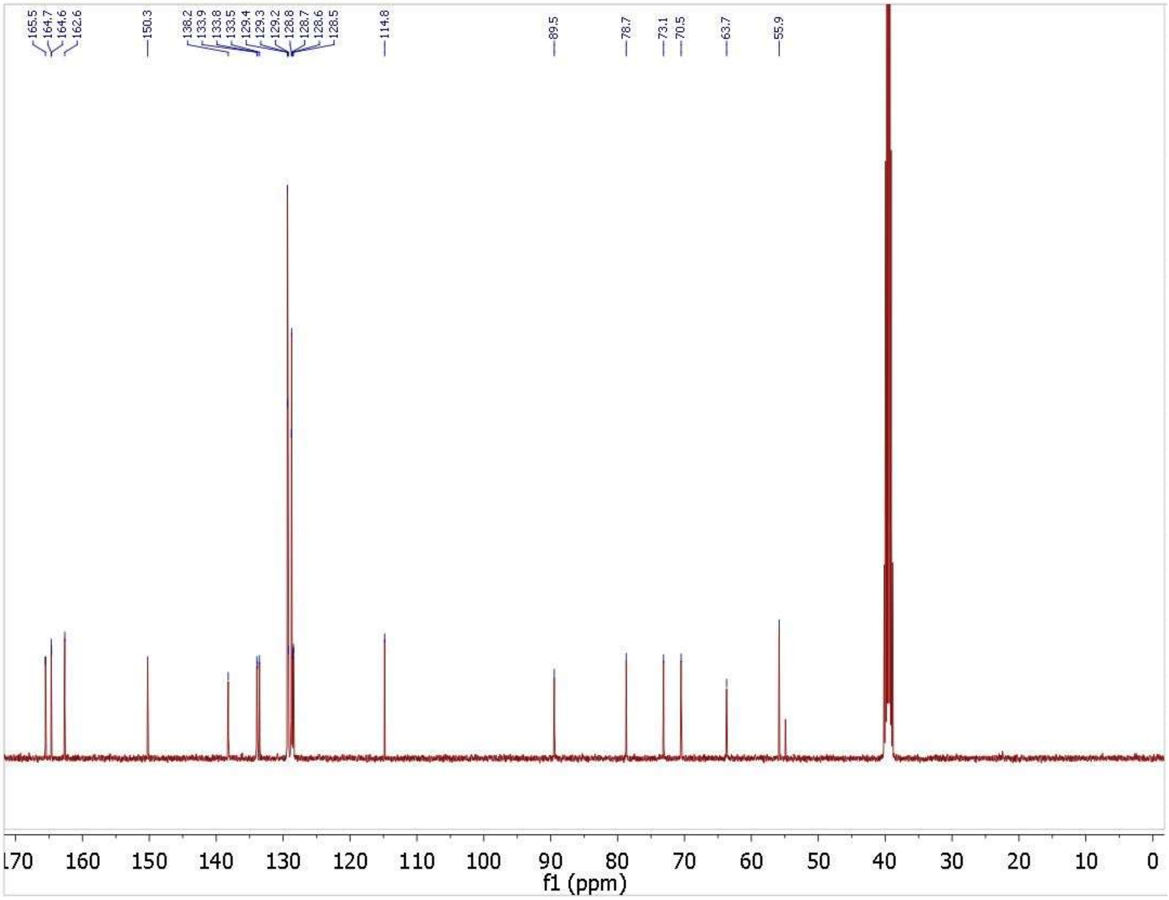

Figure S2. ^{13}C NMR spectrum (101 MHz) of **2**. Spectrum contains traces of dichloromethane (δ=54.8 ppm).

biomolecules

MDPI

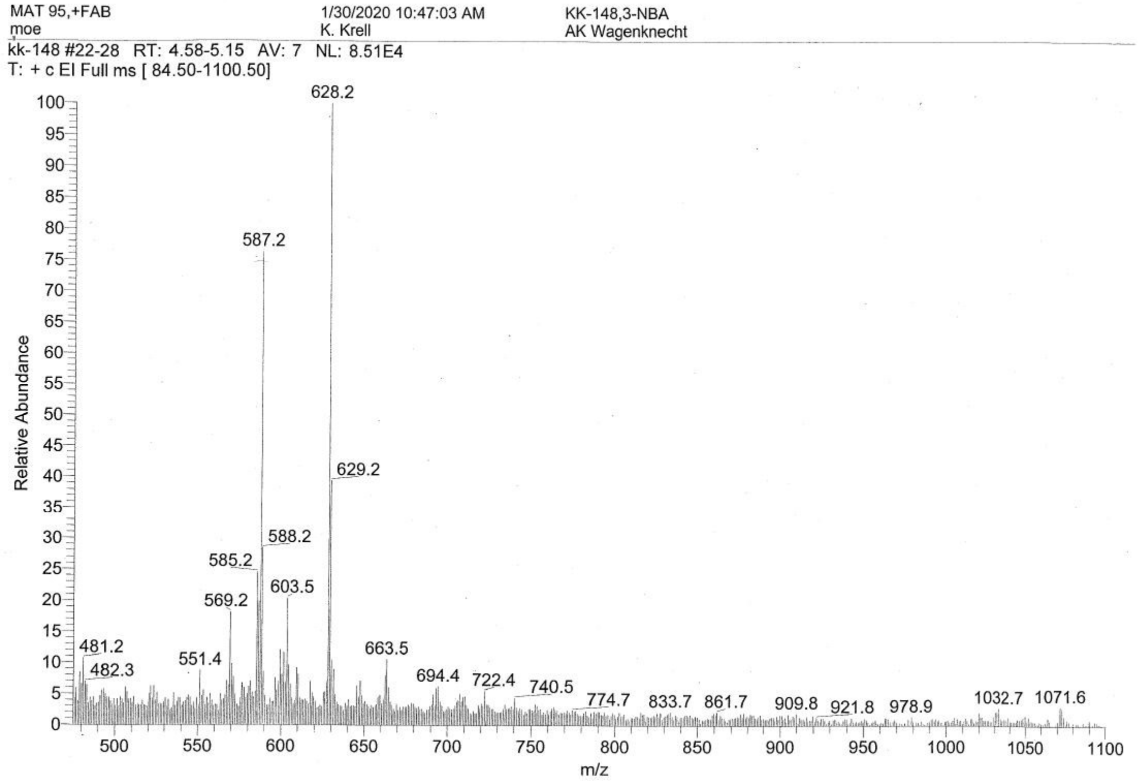

Figure S3. MS (FAB) analysis of **2**.

1/30/2020 10:56:44 AM File recalibrated by CMass.

kk-148-c4#26 RT: 4.96
T: + c EI Full ms [84.50-1100.50]
m/z= 587.0035-587.4138

m/z	Intensity	Relative	Theo. Mass	Delta (mmu)	Composition
587.1667	66998.0	100.00	587.1666	0.18	$C_{31}H_{27}O_{10}N_2$

Figure S4. HR-MS (FAB) analysis of **2**.

Compound 3

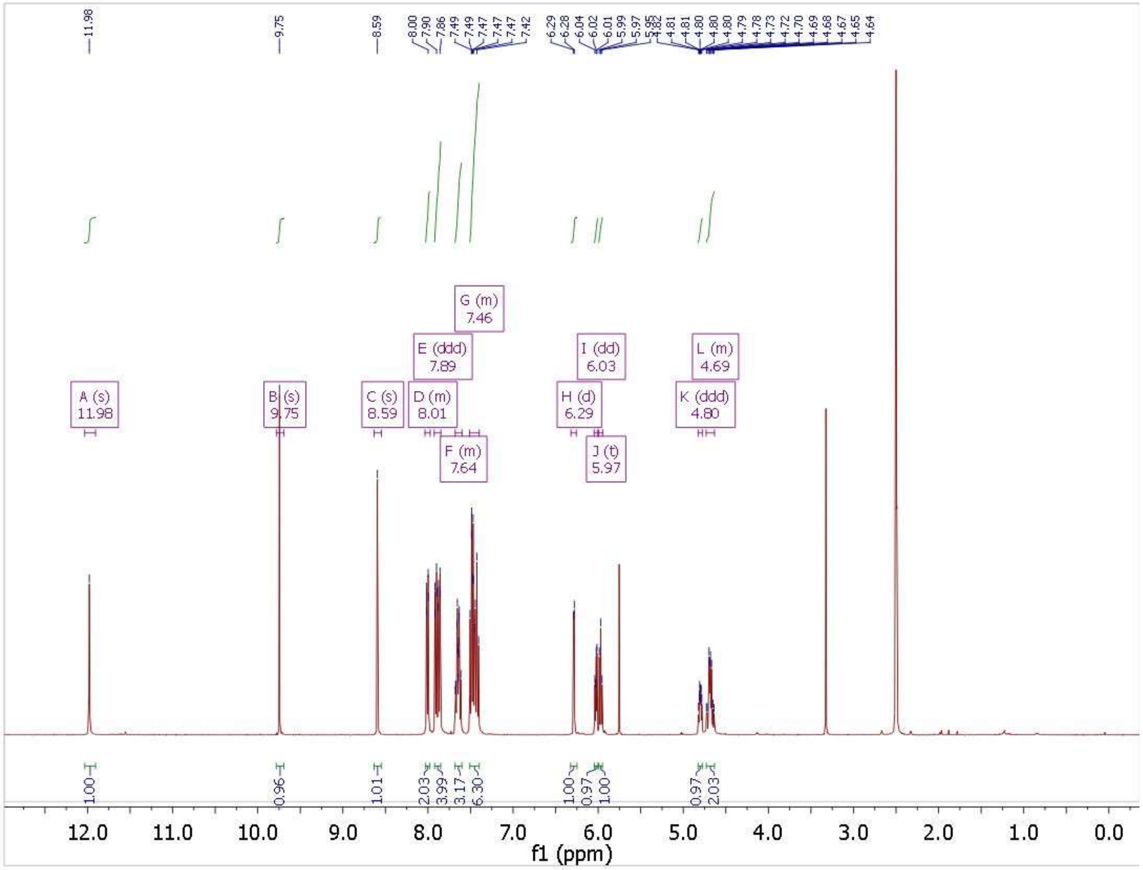

Figure S5. ^{1}H NMR spectrum (400 MHz) of **3**. Spectrum contains traces of dichloromethane (δ=5.76 ppm).

biomolecules

MDPI

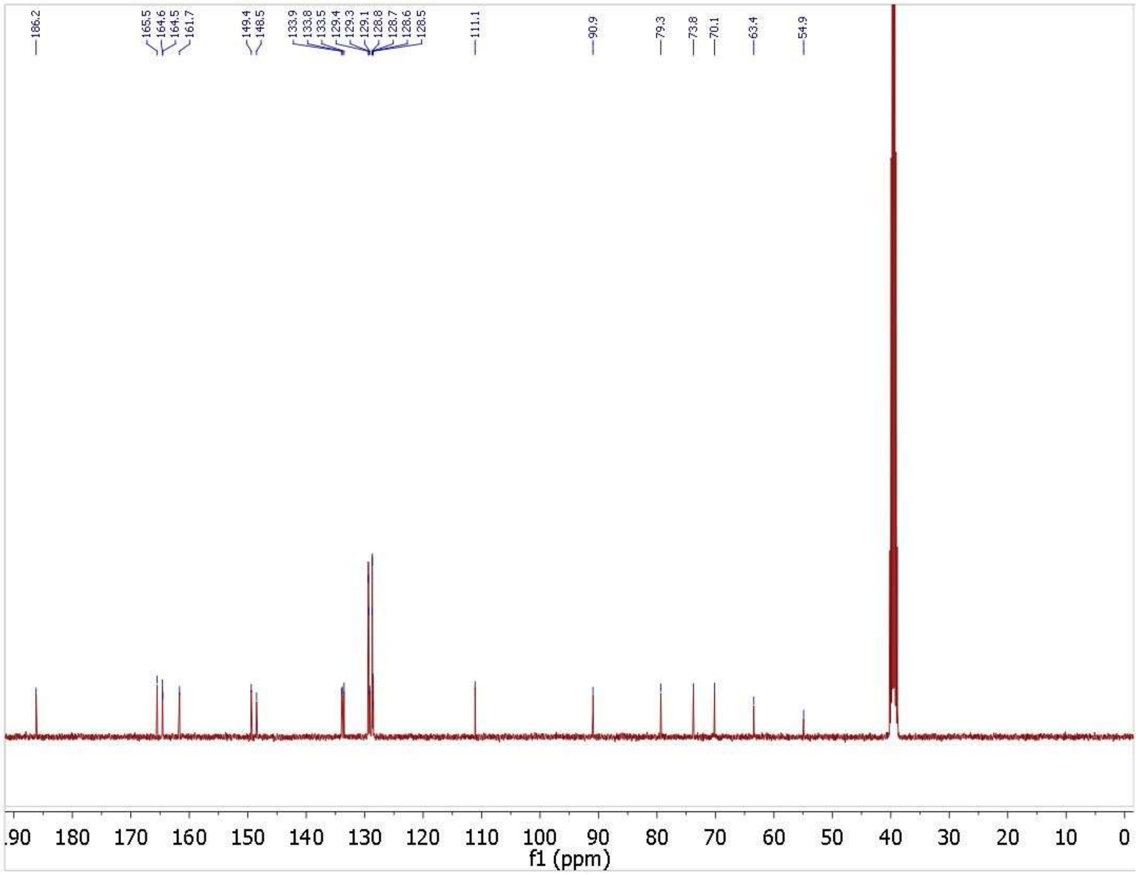

Figure S6. ^{13}C NMR spectrum (101 MHz) of **3**. Spectrum contains traces of dichloromethane (δ=54.8 ppm).

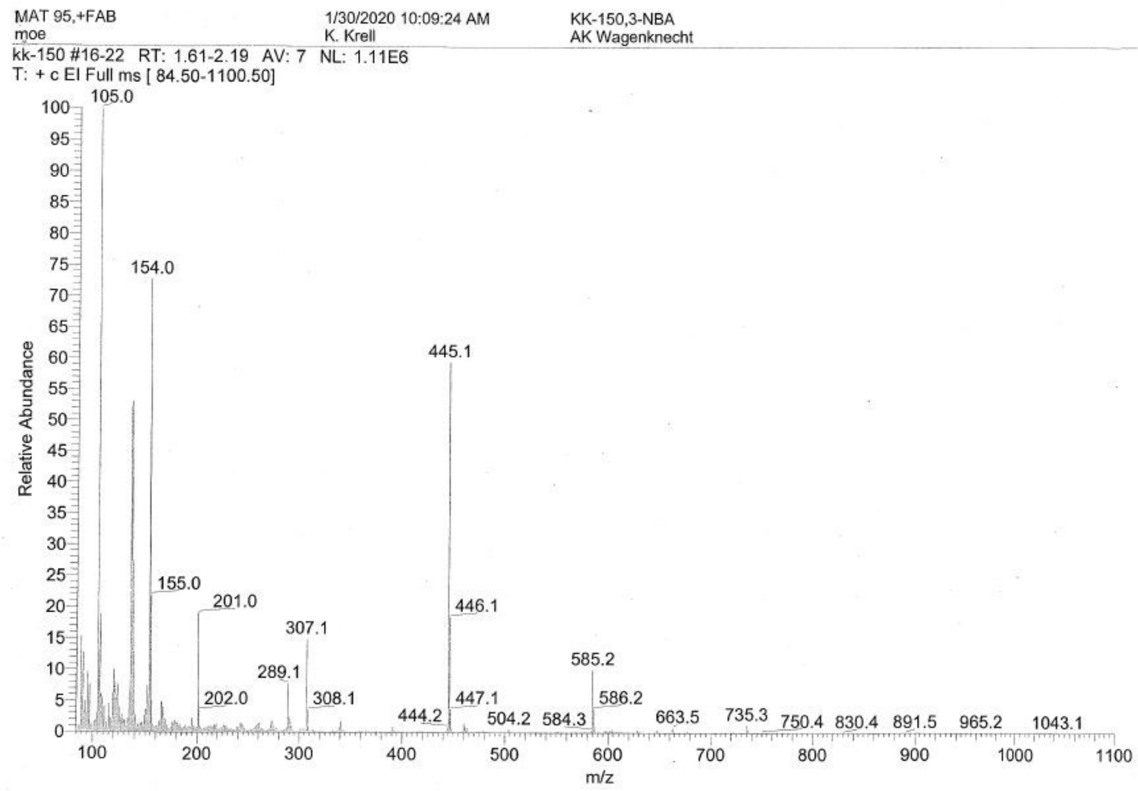

Figure S7. MS (FAB) analysis of **3**.

1/30/2020 10:19:33 AM File recalibrated by CMass.

kk-150-c1#14 RT: 1.42
T: + c EI Full ms [84.48-1100.48]
m/z= 585.1000-585.2084

m/z	Intensity	Relative	Theo. Mass	Delta (mmu)	Composition
585.1508	86929.0	100.00	585.1509	-0.16	$C_{31}H_{25}O_{10}N_2$

Figure S8. HR-MS (FAB) analysis of **3**.

biomolecules

MDPI

Compound 4

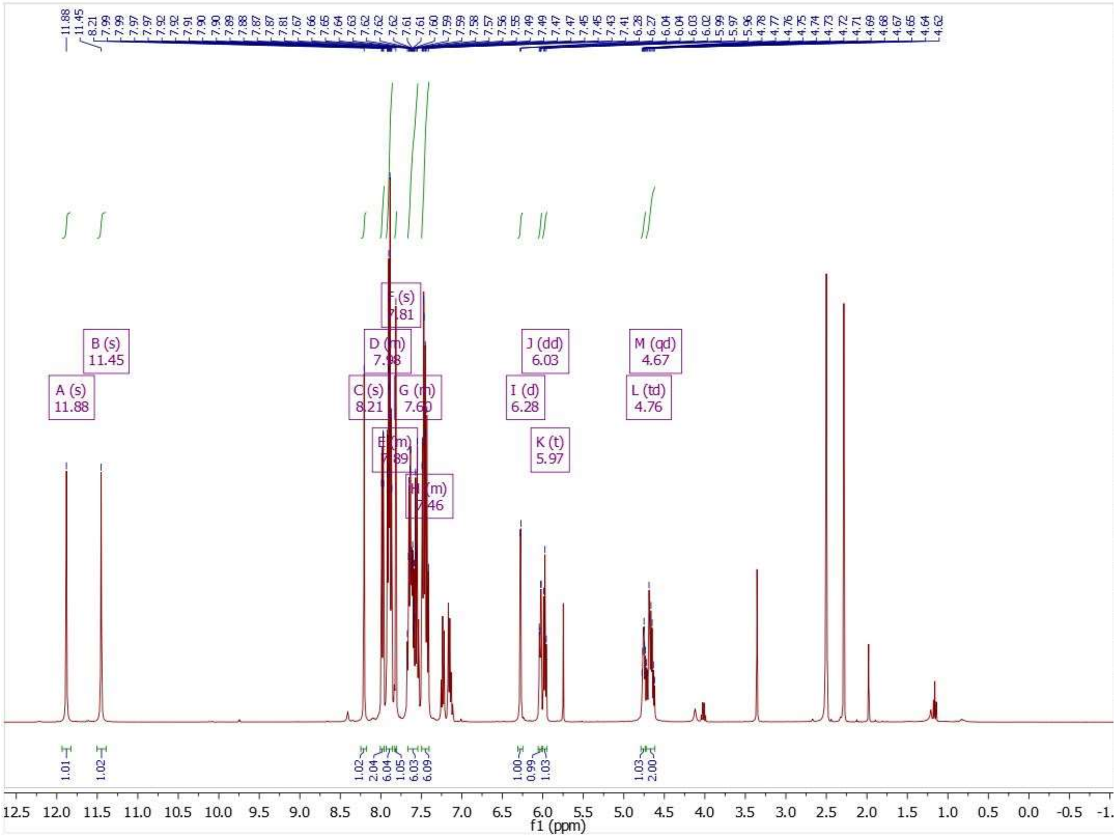

Figure S9. ^{1}H NMR spectrum (400 MHz) of **4**. Spectrum contains traces of toluene (δ=7.25 ppm, 7.18 ppm, 2.30 ppm), dichloromethane (δ = 5.76 ppm) and ethyl acetate (δ = 1.17 ppm, 4.03 ppm, 1.99 ppm).

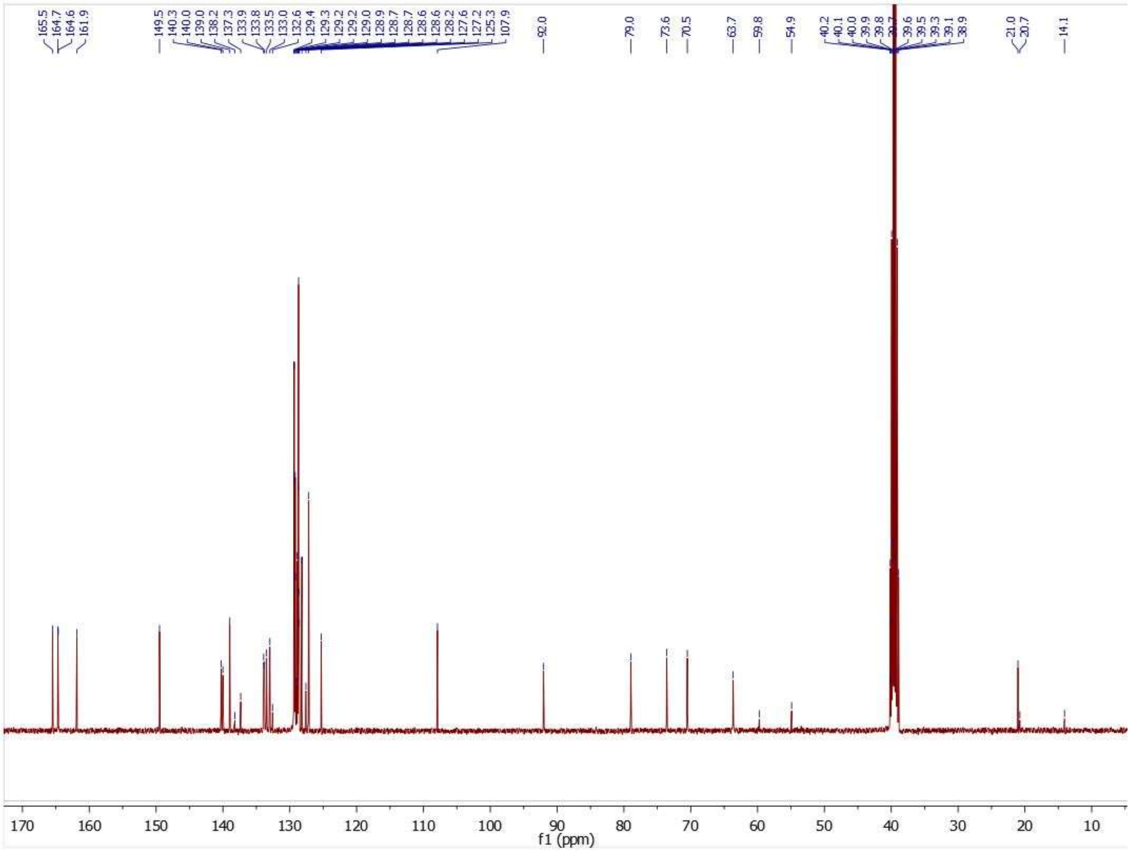

Figure S10. ^{13}C NMR spectrum (101 MHz) of **4**. Spectrum contains traces of toluene (δ = 137.4 ppm, 128.9 ppm, 128.2 ppm, 125.3 ppm, 21.0 ppm), dichloromethane (δ = 54.9 ppm) and ethyl acetate (δ=170.3 ppm, 59.8 ppm, 20.7 ppm, 14.1 ppm).

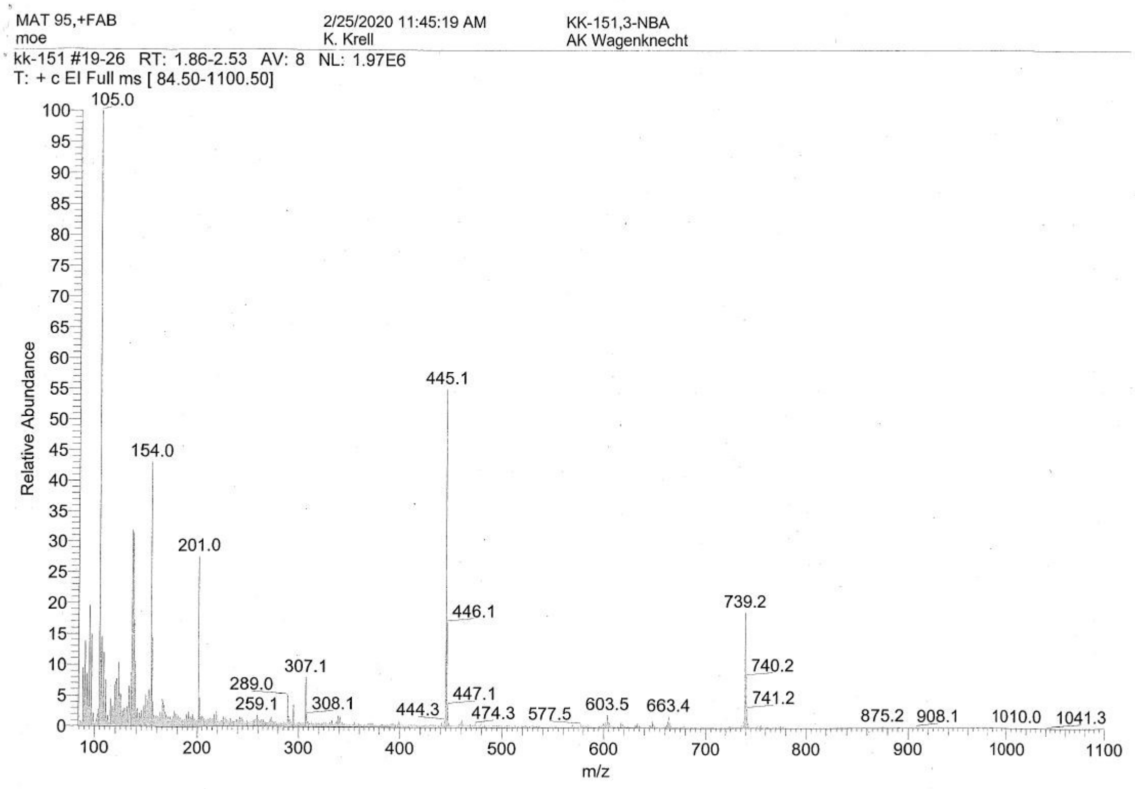

Figure S11. MS (FAB) analysis of **4**.

2/25/2020 11:54:52 AM File recalibrated by CMass.

kk-151-c5#27 RT: 2.63
T: + c EI Full ms [84.48-1100.47]
m/z= 738.8030-739.8474

m/z	Intensity	Relative	Theo. Mass	Delta (mmu)	Composition
739.1709	425007.0	100.00	739.1710	-0.07	$C_{37}H_{31}O_{11}N_4\,^{32}S_1$

Figure S12. HR-MS (FAB) analysis of **4.**

biomolecules

MDPI

Compound 5

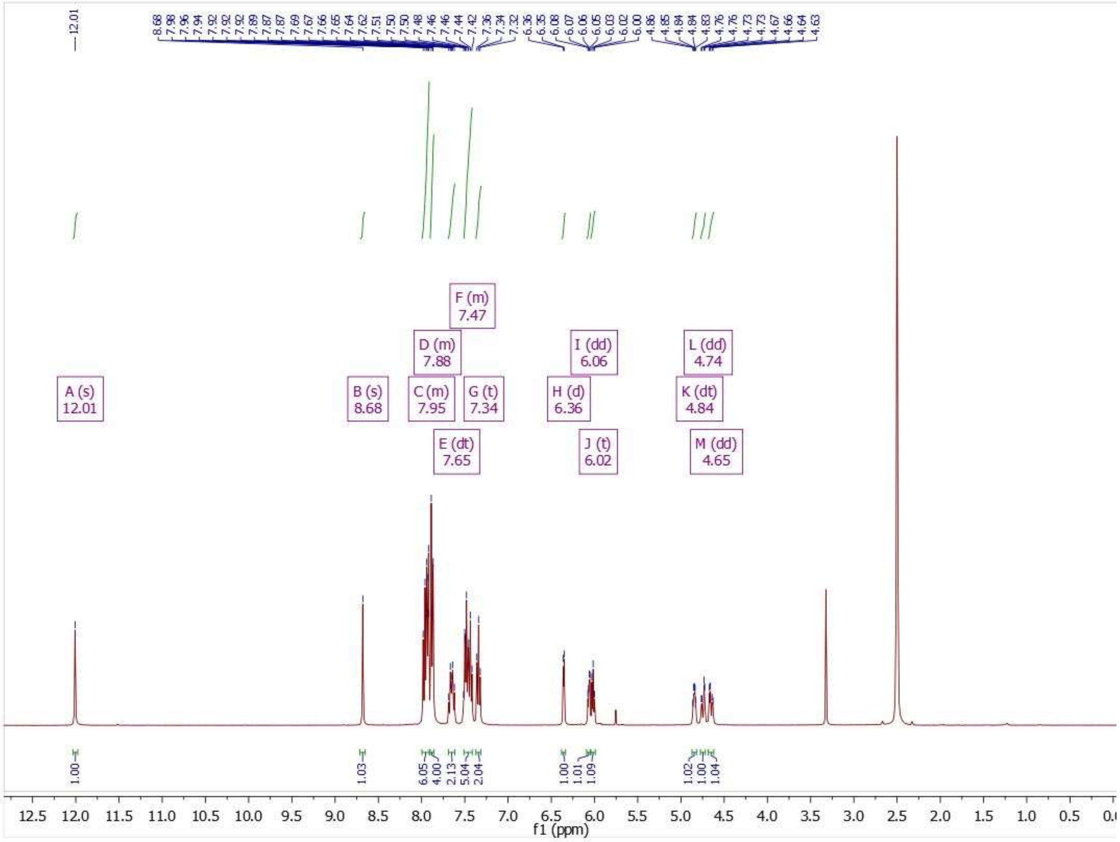

Figure S13. ^{1}H NMR spectrum (400 MHz) of **5**. Spectrum contains traces of dichloromethane (δ=5.76 ppm).

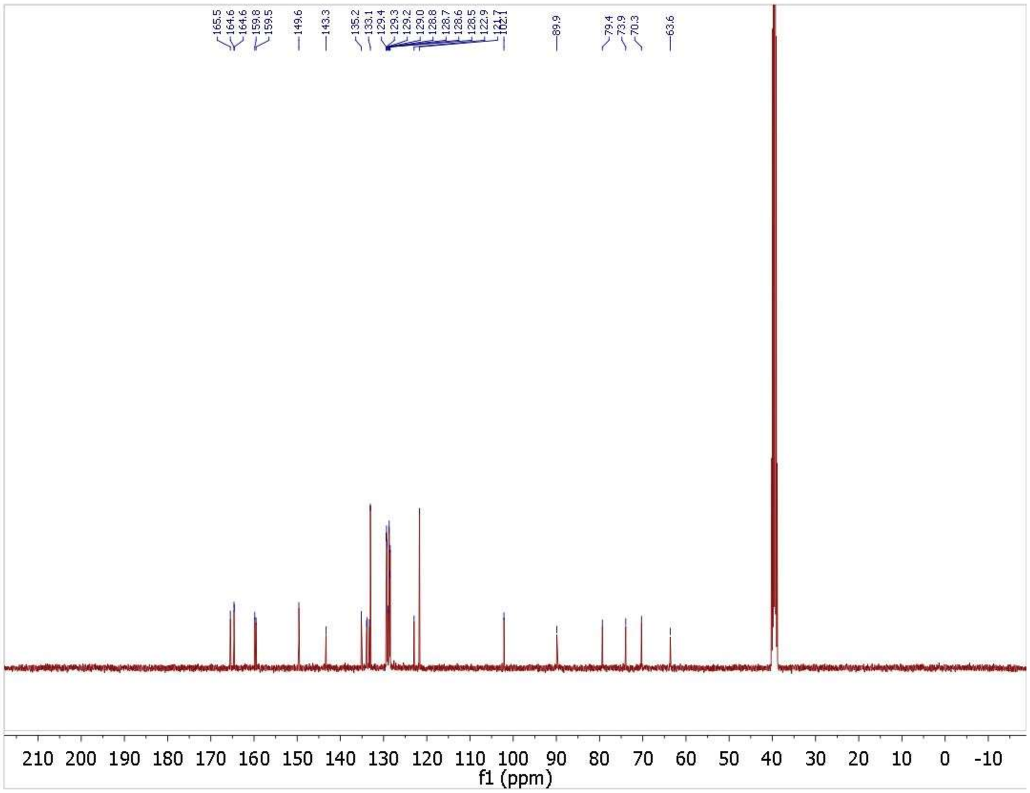

Figure S14. ^{13}C NMR spectrum (101 MHz) of **5**.

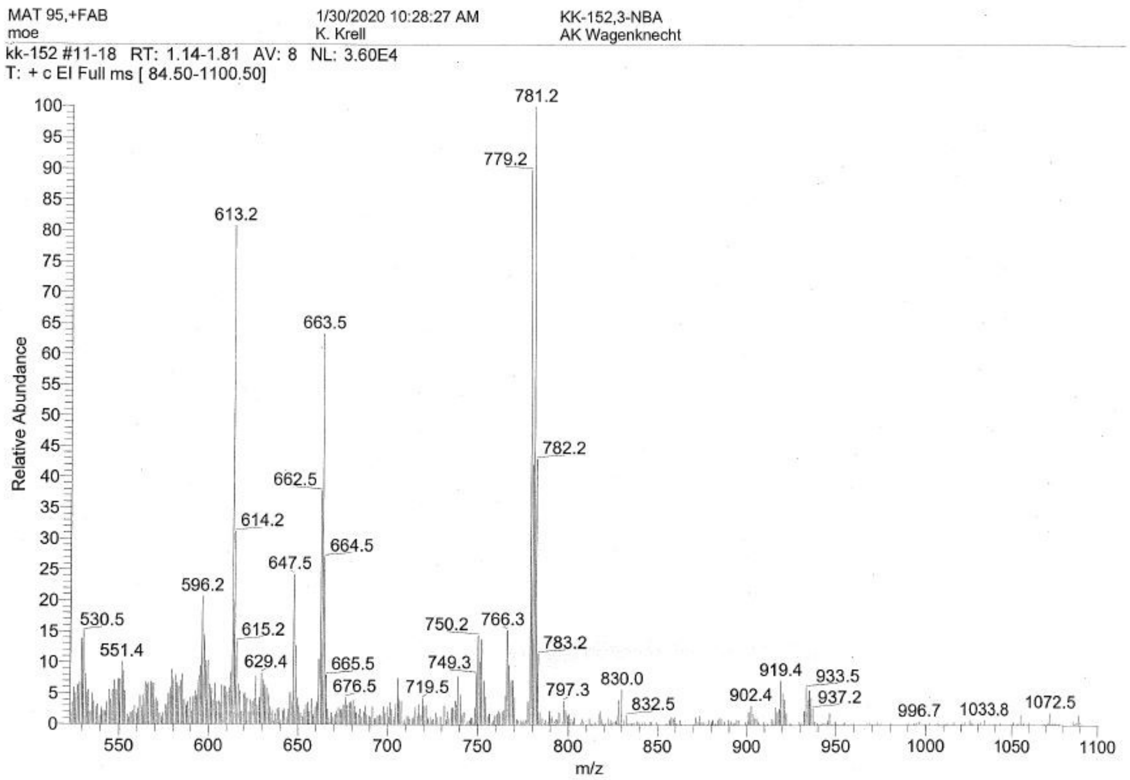

Figure S15. MS (FAB) analysis of **5**.

1/30/2020 10:41:36 AM File recalibrated by CMass.

kk-152-c9#10 RT: 1.04
T: + c EI Full ms [84.42-1100.42]
m/z= 778.9113-779.4618

m/z	Intensity	Relative	Theo. Mass	Delta (mmu)	Composition
779.1102	26609.0	100.00	779.1101	0.07	$C_{37}H_{28}O_9N_6{}^{79}Br_1$

Figure S16. HR-MS (FAB) analysis of **5**.

biomolecules

MDPI

Compound 6

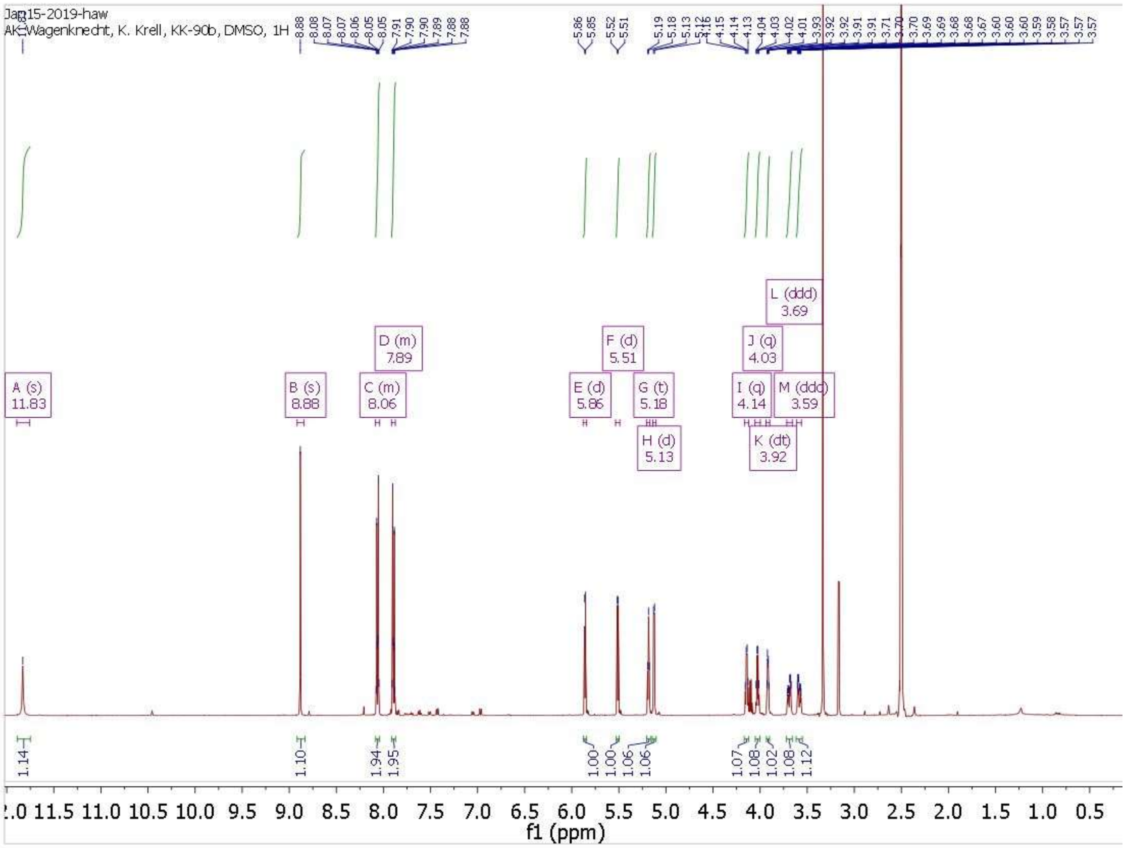

Figure S17. ^{1}H NMR spectrum (500 MHz) of **6**. The spectrum contains traces of methanol (δ=4.01 ppm, 3.16 ppm).

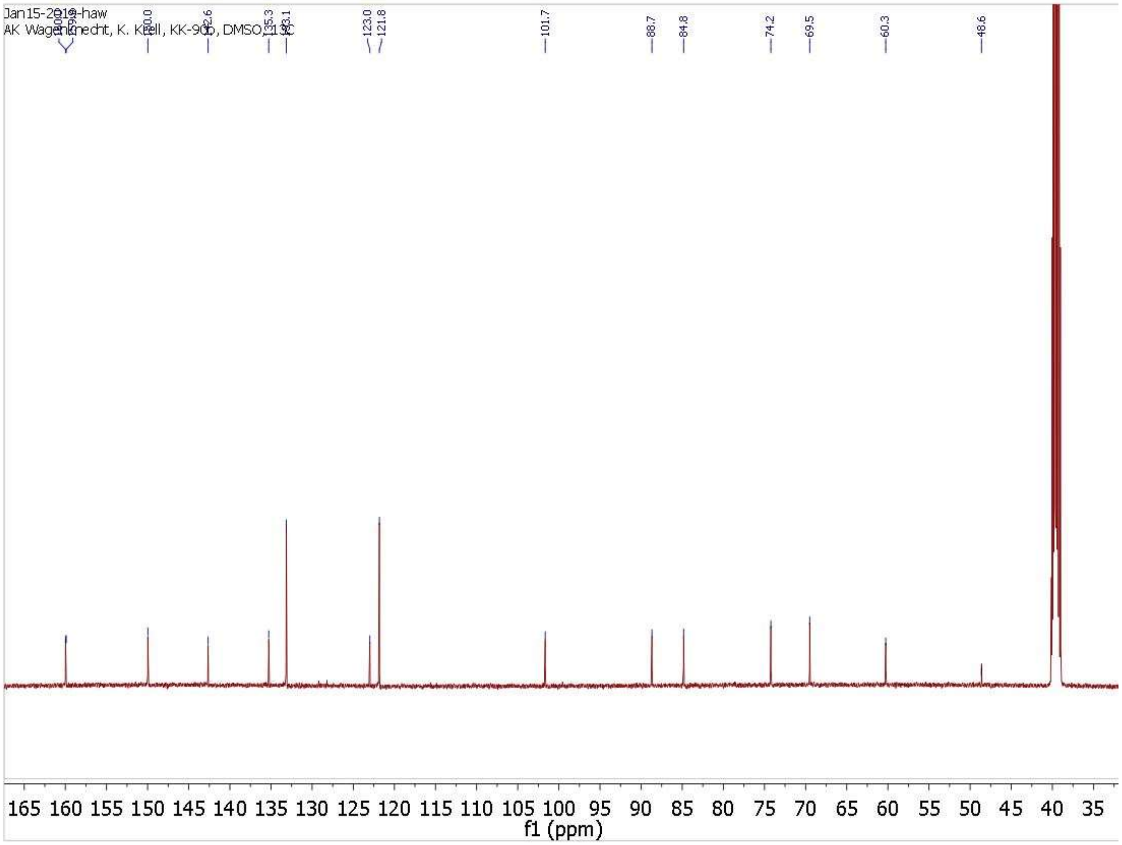

Figure S18. ^{13}C NMR spectrum (126 MHz) of **6**. The spectrum contains traces of methanol (δ=48.6 ppm).

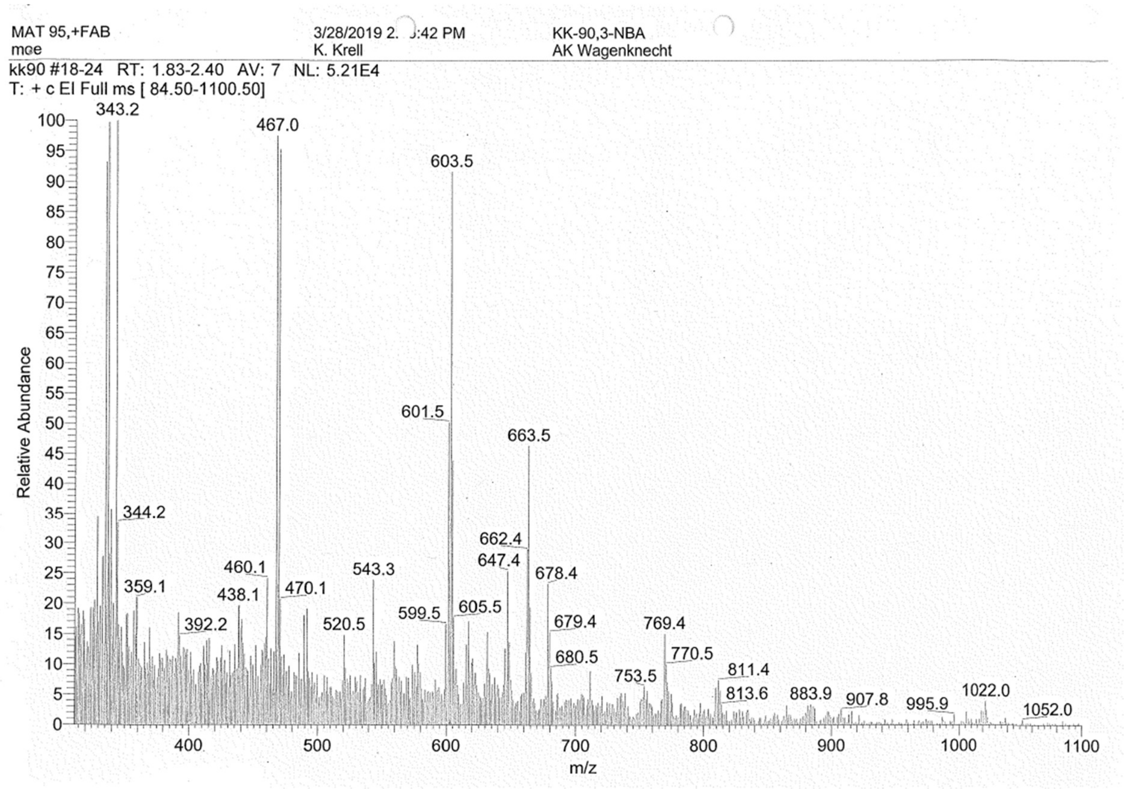

Figure S19. MS (FAB) analysis of **6**.

3/28/2019 2:31:16 PM File recalibrated by CMass.

kk90-c2#25 RT: 2.50
T: + c EI Full ms [84.52-1100.52]
m/z= 466.9708-467.0833

m/z	Intensity	Relative	Theo. Mass	Delta (mmu)	Composition
467.0314	53842.0	100.00	467.0315	-0.04	$C_{16}H_{16}O_{6}N_{6}{}^{79}Br_{1}$

Figure S20. HR-MS (FAB) analysis of **6**.

biomolecules

MDPI

Compound 7

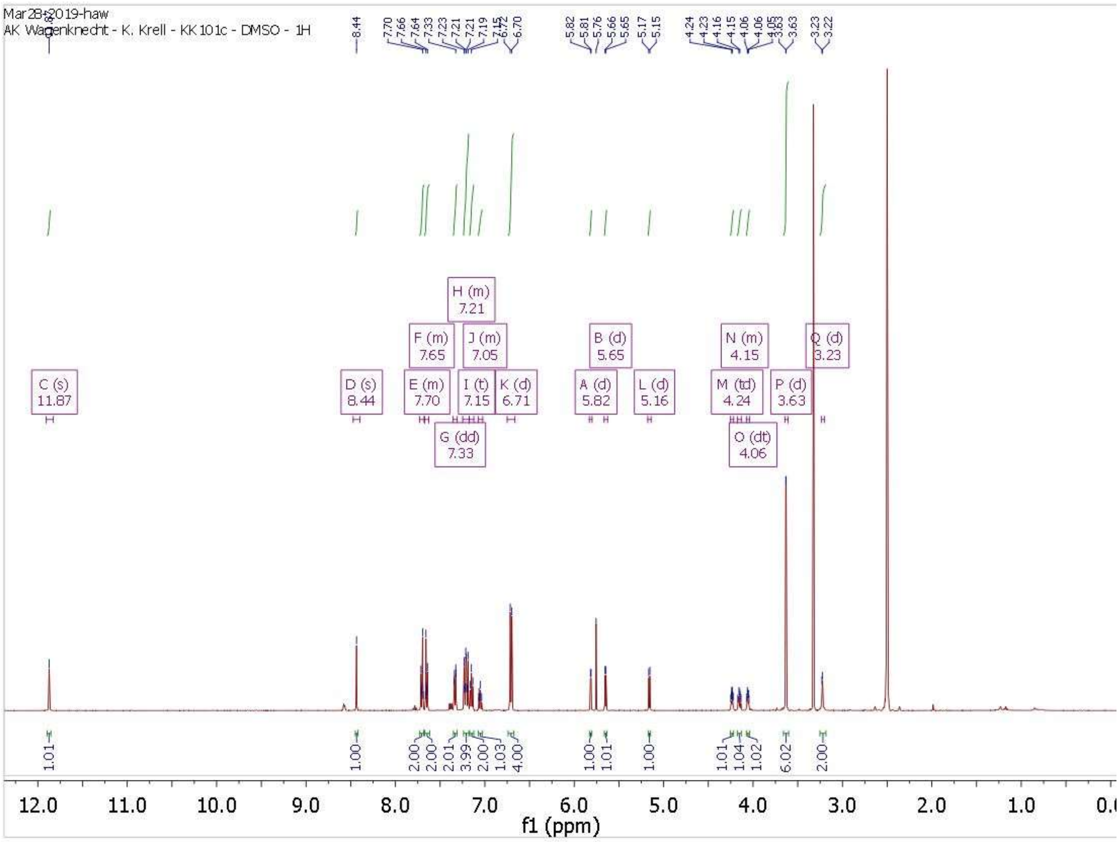

Figure S21. ^{1}H NMR spectrum (500 MHz) of **7**. Spectrum contains traces of dichloromethane (δ=5.76ppm).

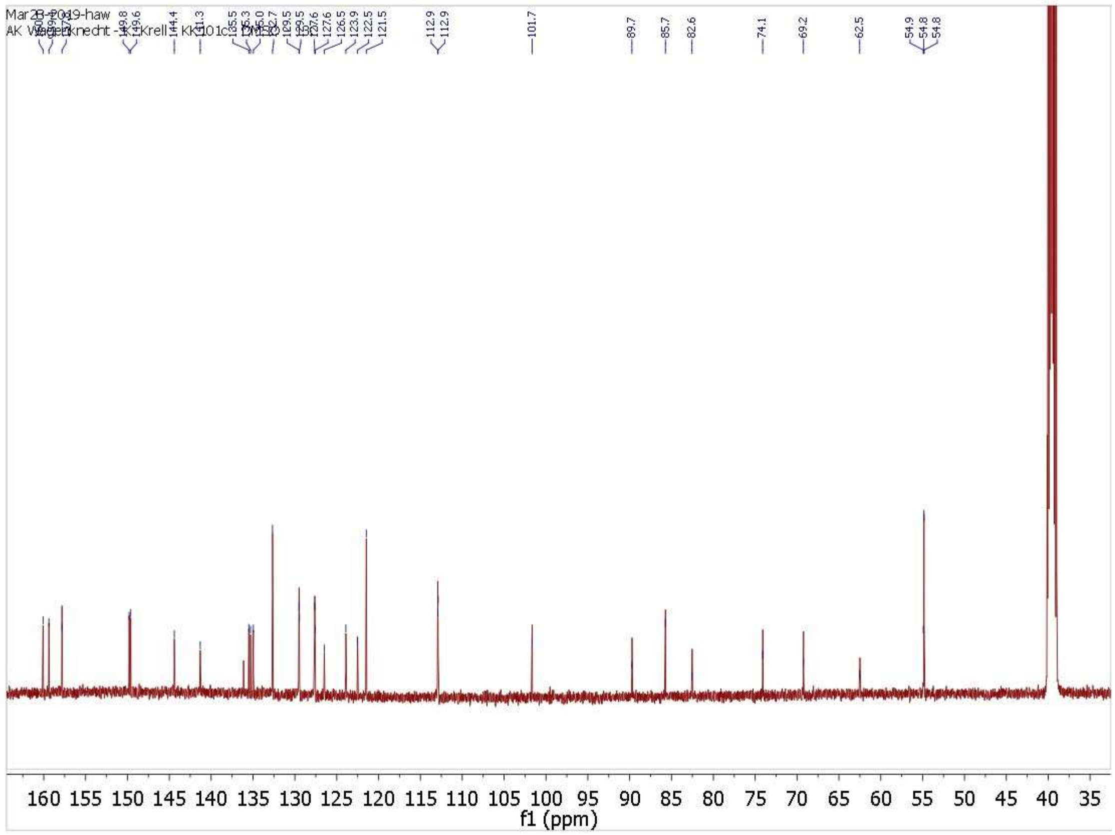

Figure S22. ^{13}C NMR spectrum (126 MHz) of **7**. Spectrum contains traces of dichloromethane (δ=54.9 ppm).

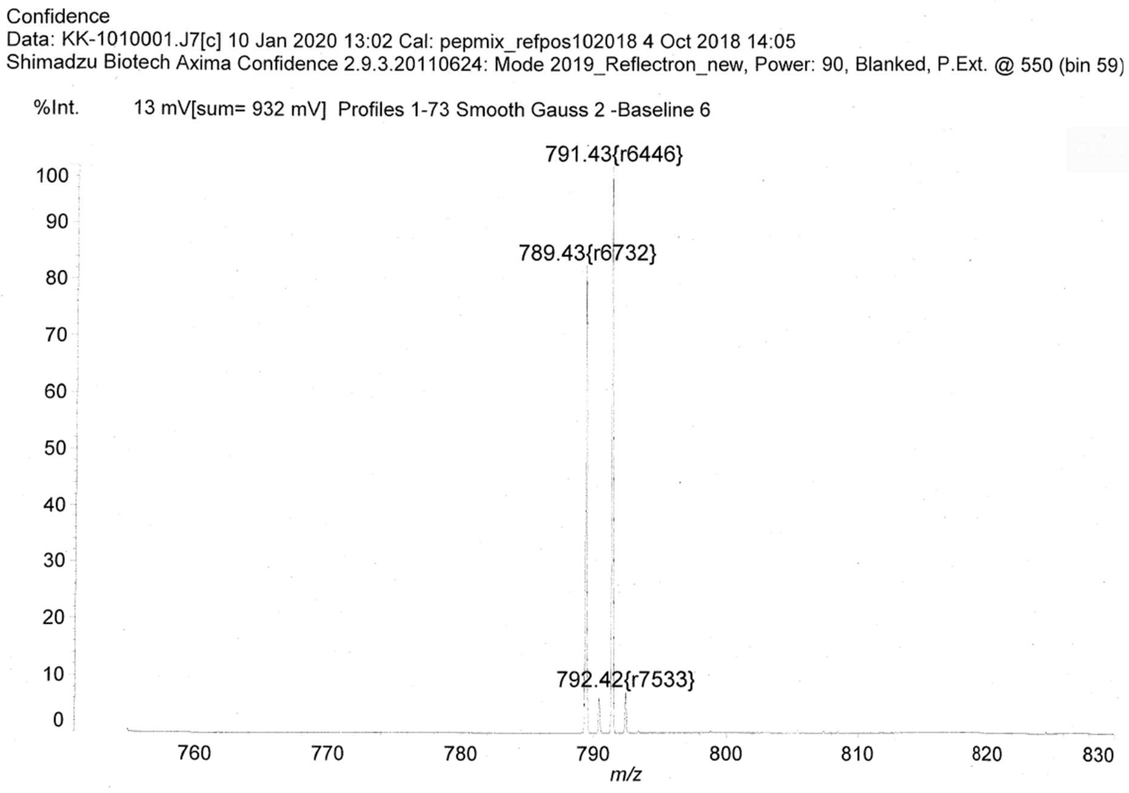

Figure S23. MS (MALDI-TOF) analysis of **7**.

Compound 8

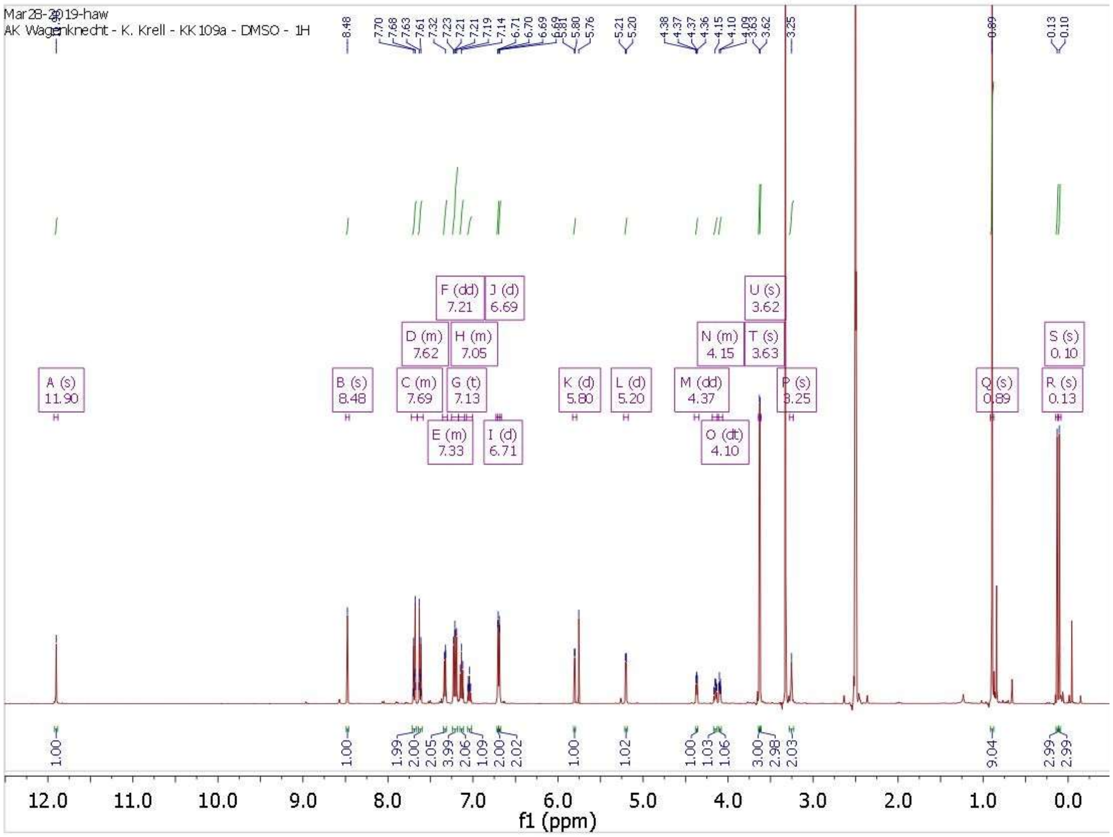

Figure S24. ^{1}H NMR spectrum (500 MHz) of **8**. Spectrum contains traces of dichloromethane (δ=5.76 ppm).

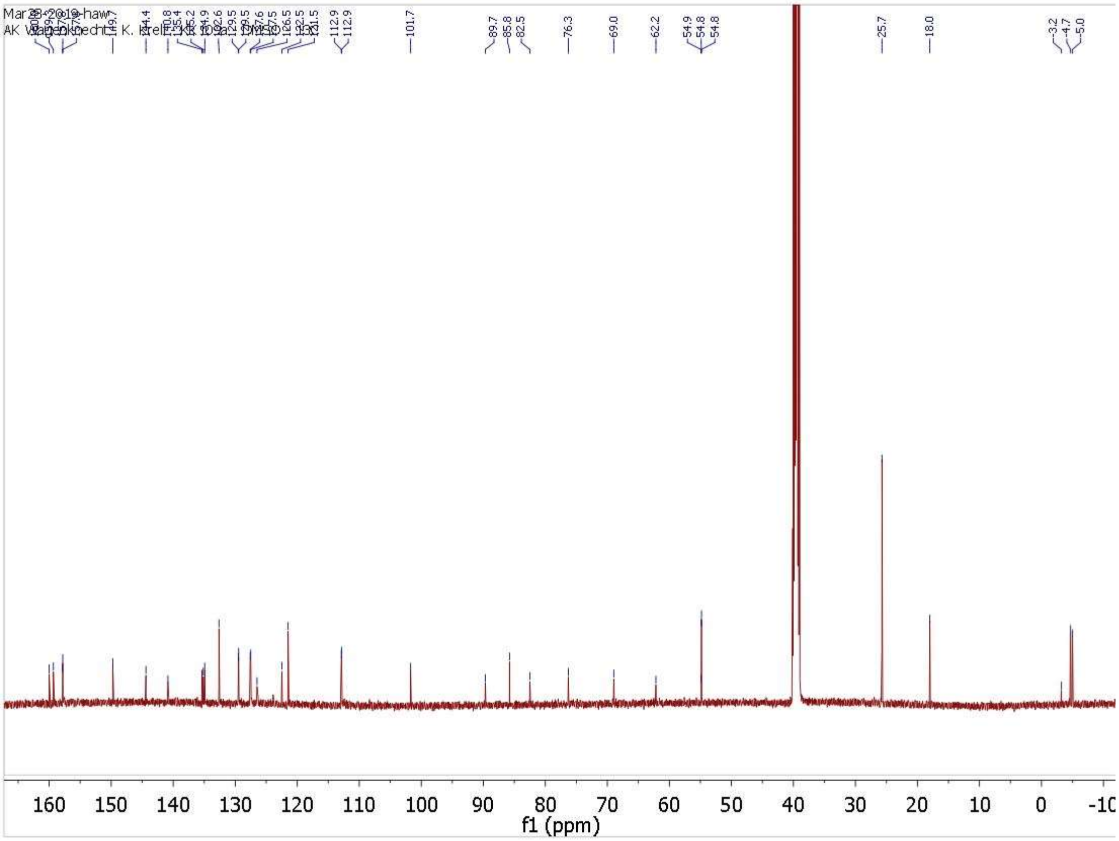

Figure S25. ^{13}C NMR spectrum (126 MHz) of **8**. Spectrum contains traces of dichloromethane (δ=54.9 ppm).

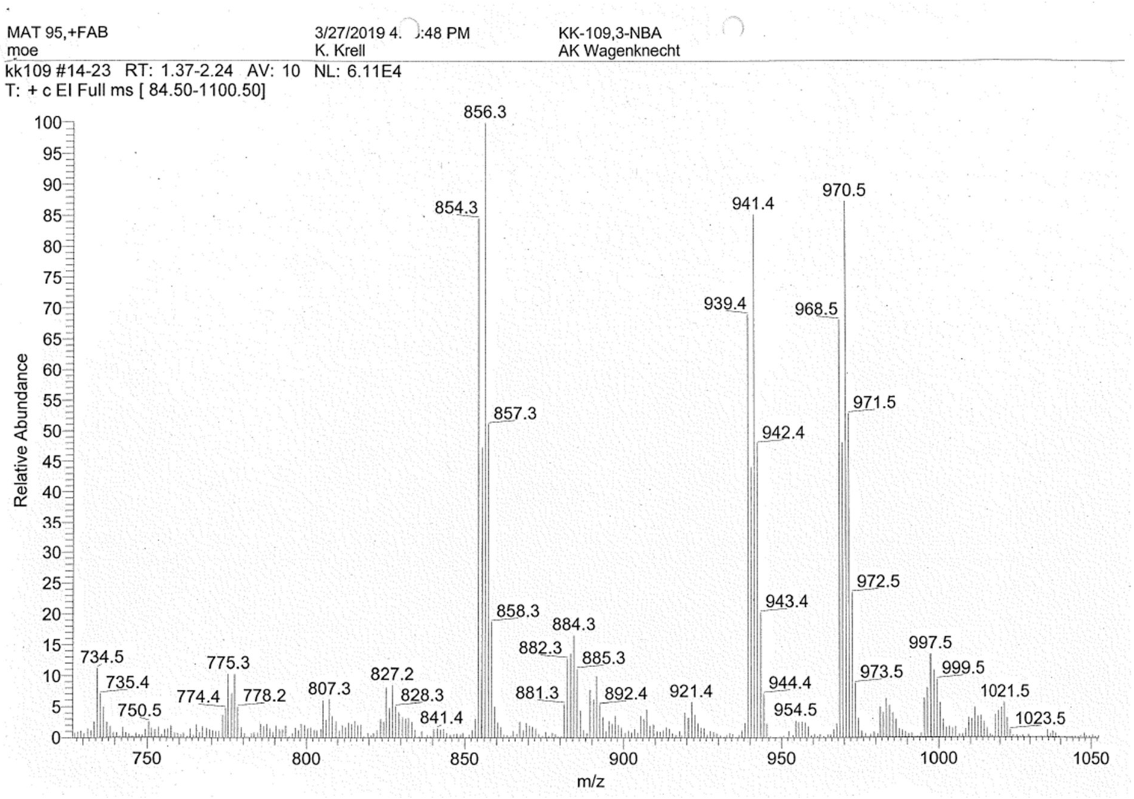

Figure S26. MS (FAB) analysis of **8**.

3/27/2019 4:29:11 PM File recalibrated by CMass.

kk109-c1#15 RT: 1.47
T: + c EI Full ms [84.42-1100.42]
m/z= 881.9756-882.6086

m/z	Intensity	Relative	Theo. Mass	Delta (mmu)	Composition
882.2411	6889.0	100.00	882.2408	0.30	$C_{43}H_{47}O_{8}N_{6}{}^{79}Br_{1}{}^{28}Si_{1}$

Figure S27. HR-MS (FAB) analysis of **8**.

biomolecules

MDPI

Compound 9

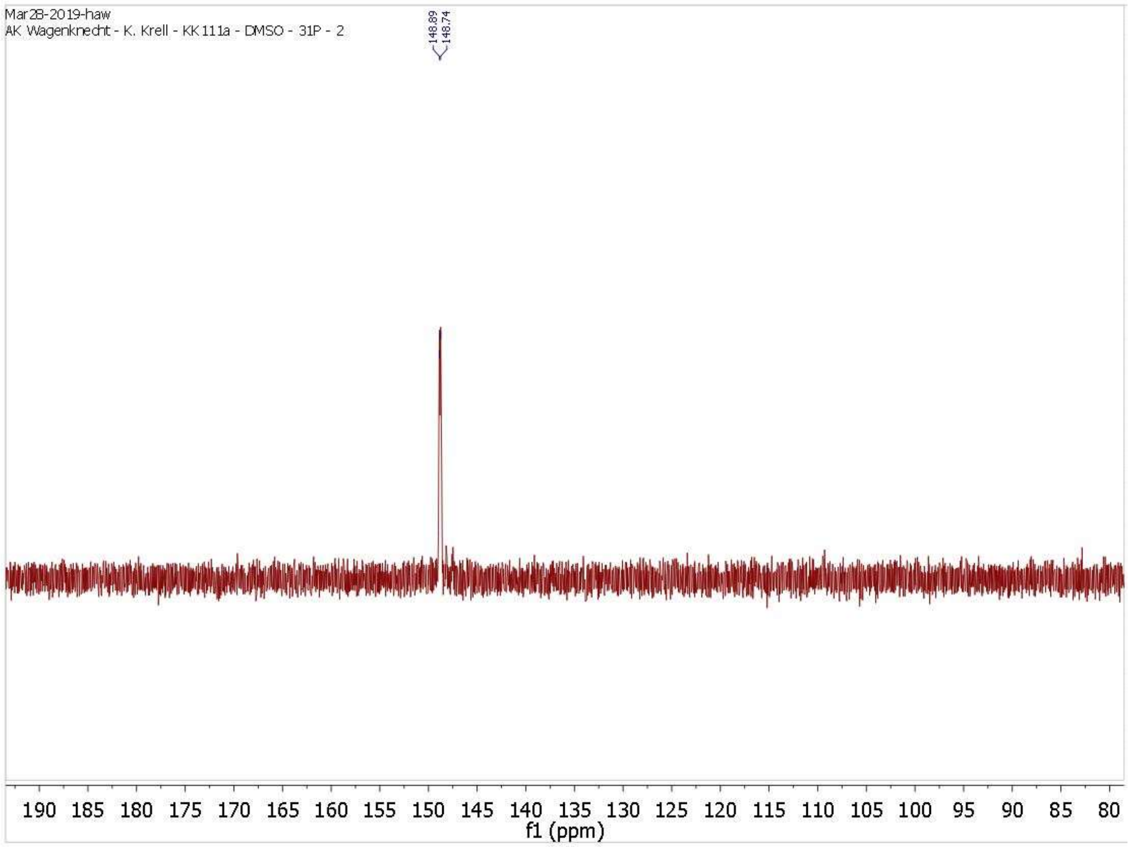

Figure S28. ^{31}P NMR spectrum (202 MHz) of **9**.

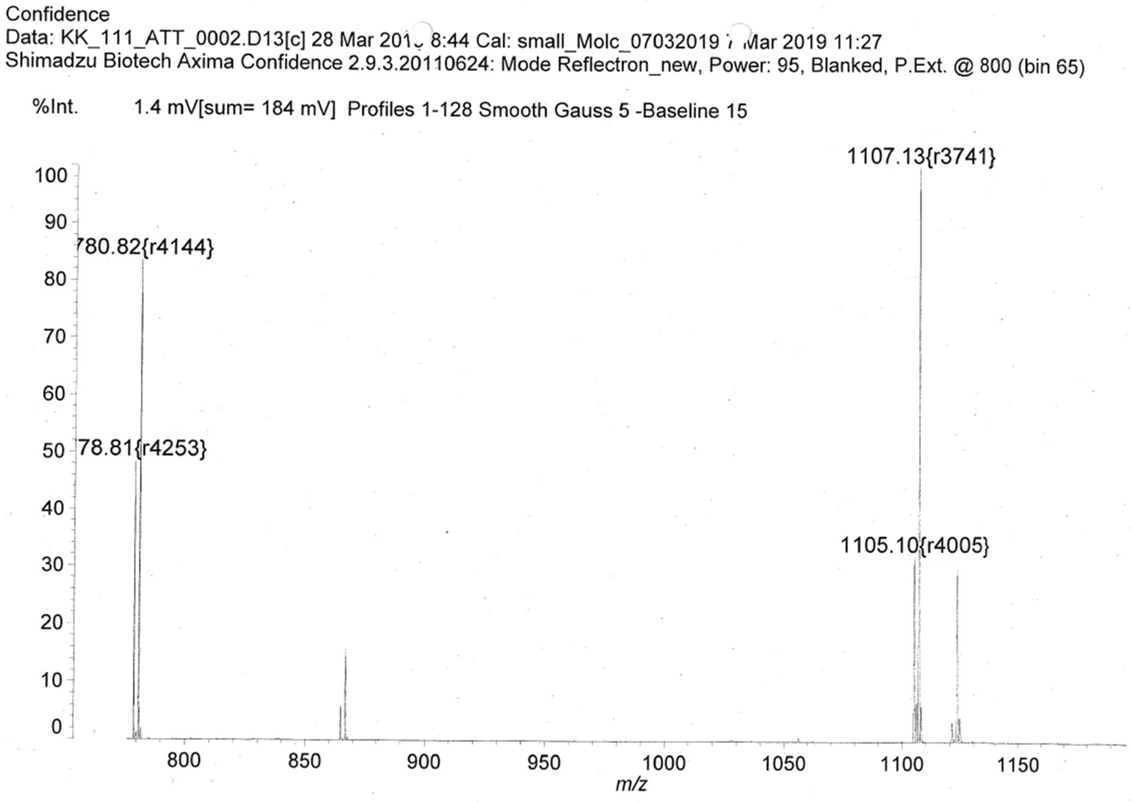

Figure S29. MS (MALDI-TOF) analysis of **9**.

2. Optical Spectroscopy

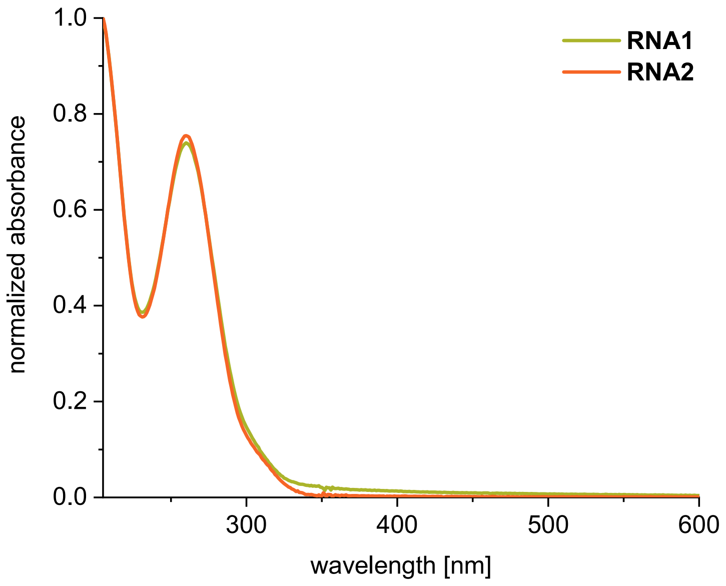

Figure S30. UV/Vis absorbance of **RNA1** and **RNA2** (2.5 µM) in 10 mM Na-P$_i$ buffer, 250 mM NaCl, pH 7. The spectra were normalized to evaluate the relative tetrazole absorbances.

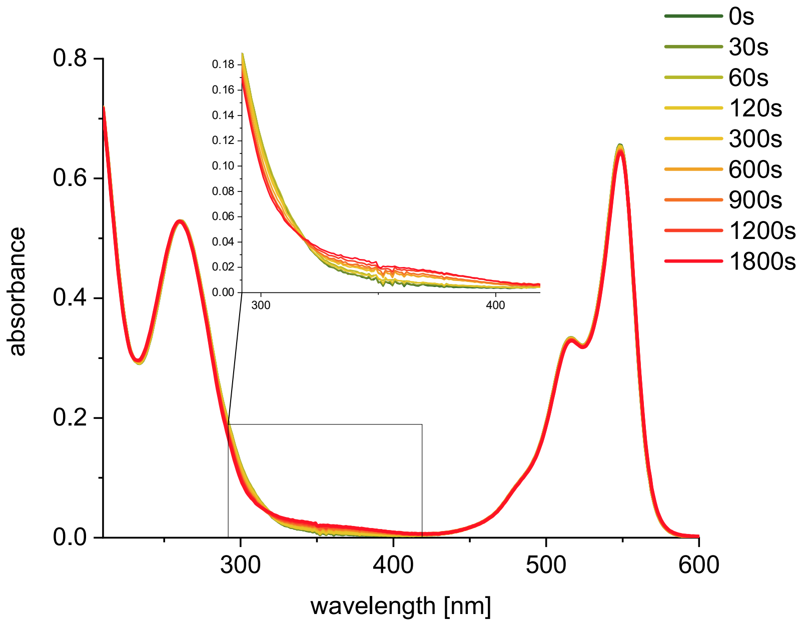

Figure S31. UV/Vis absorbance recorded during reaction of **RNA1** (2.5 µM) with Cy3-maleimide (3.75 µM, 1.50 equiv.), irradiated at 300 nm (LED) in 10 mM Na-P$_i$ buffer, 250 mM NaCl, pH 7.

biomolecules

MDPI

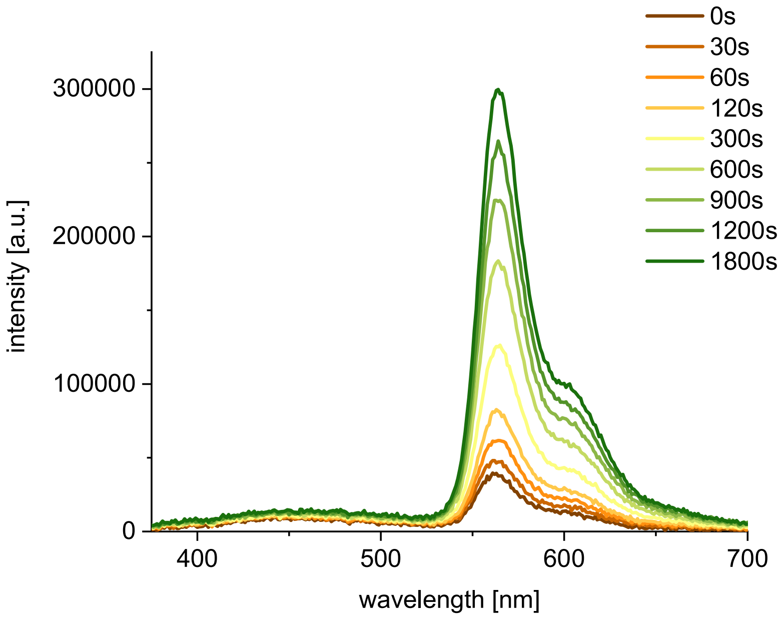

Figure S32. Fluorescence recorded during reaction of **RNA1** (2.5 µM) with Cy3-maleimide (3.75 µM, (1.50 equiv.), irradiated at 300 nm (LED) in 10 mM Na-P_i buffer, 250 mM NaCl, pH 7. Fluorescence excitation at 358 nm.

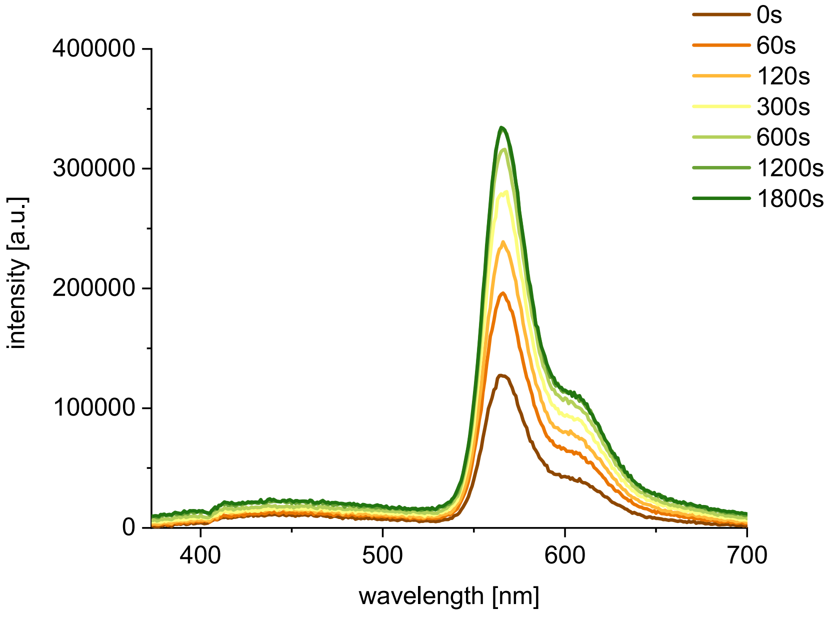

Figure S33. Fluorescence recorded during reaction of **RNA1** (2.5 µM) with AF555-maleimide (3.75 µM, (1.50 equiv.), irradiated at 300 nm (LED) in 10 mM Na-P_i buffer, 250 mM NaCl, pH 7. Fluorescence excitation at 358 nm.

biomolecules

MDPI

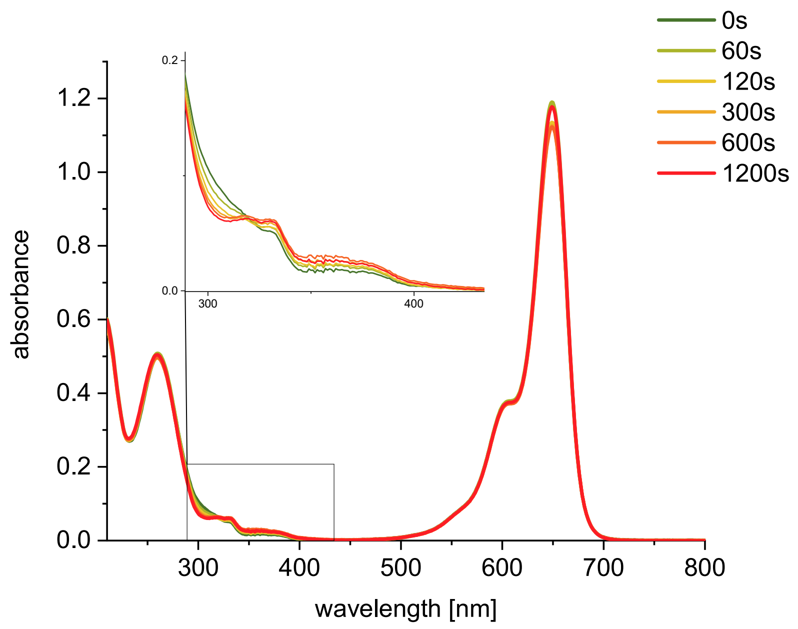

Figure S34. UV/Vis absorbance recorded during reaction of **RNA1** (2.5 µM) with AF647-maleimide (3.75 µM, 1.50 equiv.), irradiated at 300 nm (LED) in 10 mM Na-P_i buffer, 250 mM NaCl, pH 7.

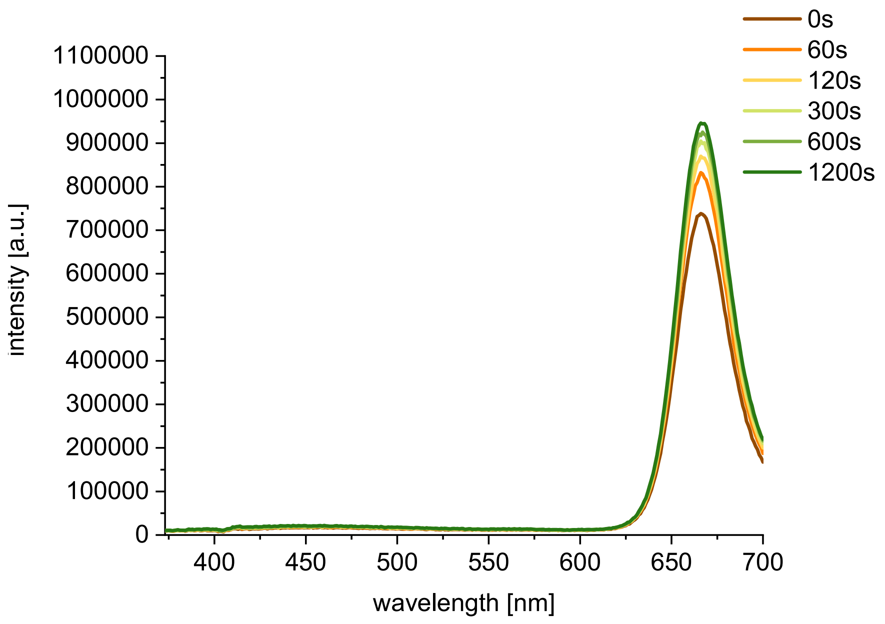

Figure S35. Fluorescence recorded during reaction of **RNA1** (2.5 µM) with AF647-maleimide (3.75 µM, 1.50 eq), irradiated at 300 nm (LED) in 10 mM Na-P_i buffer, 250 mM NaCl, pH 7. Fluorescence excitation at 358 nm.

biomolecules

MDPI

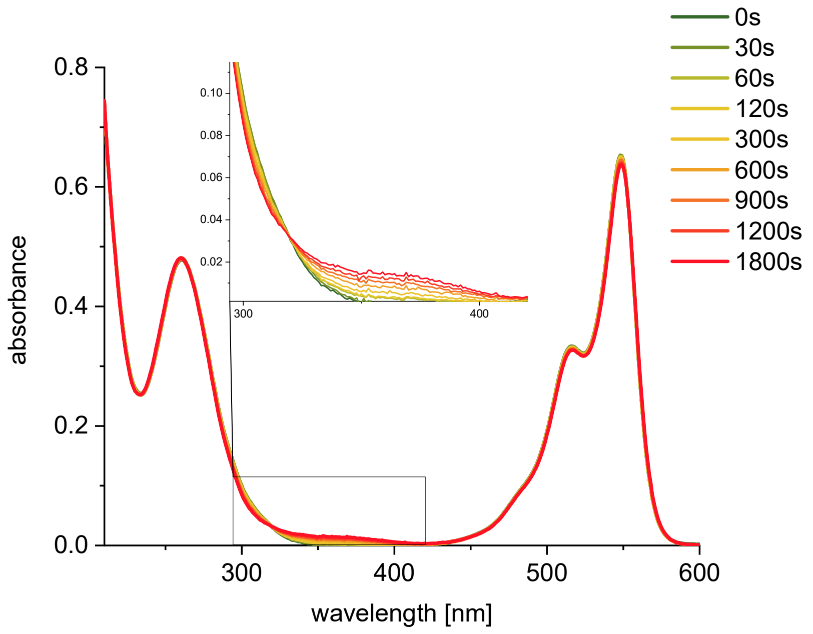

Figure S36. UV/vis absorbance recorded during reaction of **RNA2** (2.5 µM) with Cy3-maleimide (3.75 µM, 1.50 equiv.), irradiated at 300 nm (LED) in 10 mM Na-P_i buffer, 250 mM NaCl, pH 7.

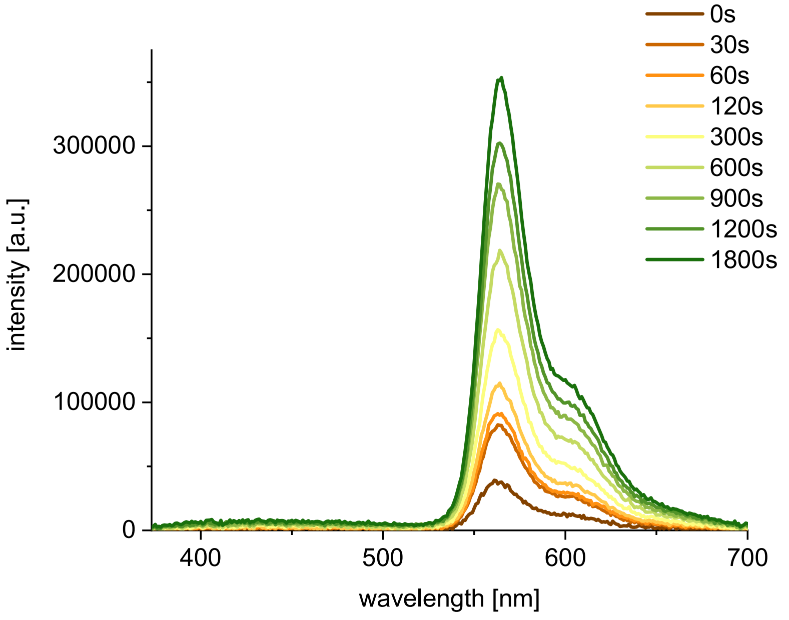

Figure S37. Fluorescence recorded during reaction of **RNA2** (2.5 µM) with Cy3-maleimide (3.75 µM, 1.50 equiv.), irradiated at 300 nm (LED) in 10 mM Na-P_i buffer, 250 mM NaCl, pH 7. Fluorescence excitation at 358 nm.

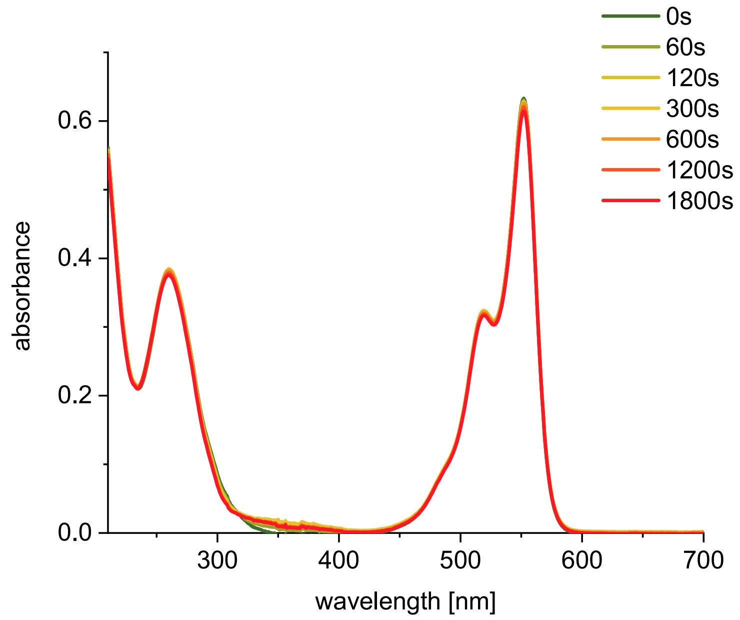

Figure S38. UV/Vis absorbance recorded during reaction of **RNA2** (2.5 µM) with AF555-maleimide (3.75 µM, 1.50 equiv.), irradiated at 300 nm (LED) in 10 mM Na-P_i buffer, 250 mM NaCl, pH 7.

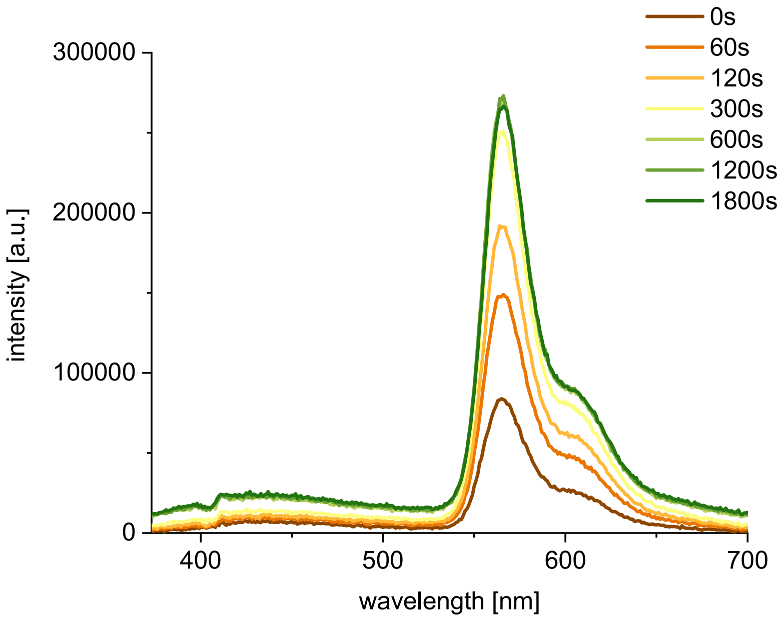

Figure S39. Fluorescence recorded during reaction of **RNA2** (2.5 µM) with AF555-maleimide (3.75 µM, 1.50 equiv.), irradiated at 300 nm (LED) in 10 mM Na-P_i buffer, 250 mM NaCl, pH 7. Fluorescence excitation at 358 nm.

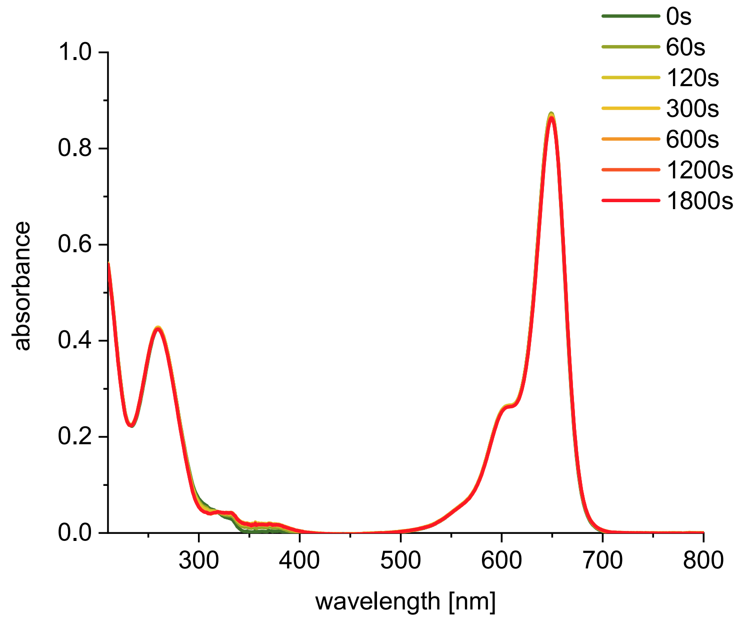

Figure S40. UV/Vis absorbance recorded during reaction of **RNA2** (2.5 µM) with AF647-maleimide (3.75 µM, 1.50 equiv.), irradiated at 300 nm (LED) in 10 mM Na-P_i buffer, 250 mM NaCl, pH 7.

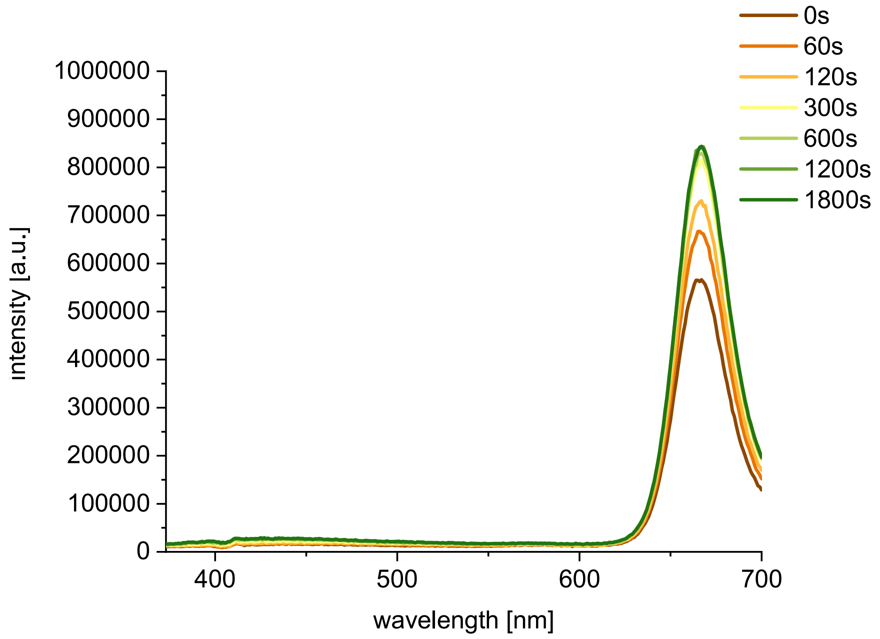

Figure S41. Fluorescence recorded during reaction of **RNA2** (2.5 µM) with AF647-maleimide (3.75 µM, 1.50 equiv.), irradiated at 300 nm (LED) in 10 mM Na-P_i buffer, 250 mM NaCl at pH 7. Fluorescence excitation at 358 nm.

biomolecules

MDPI

3. MALDI spectra of RNA strands

RNA1

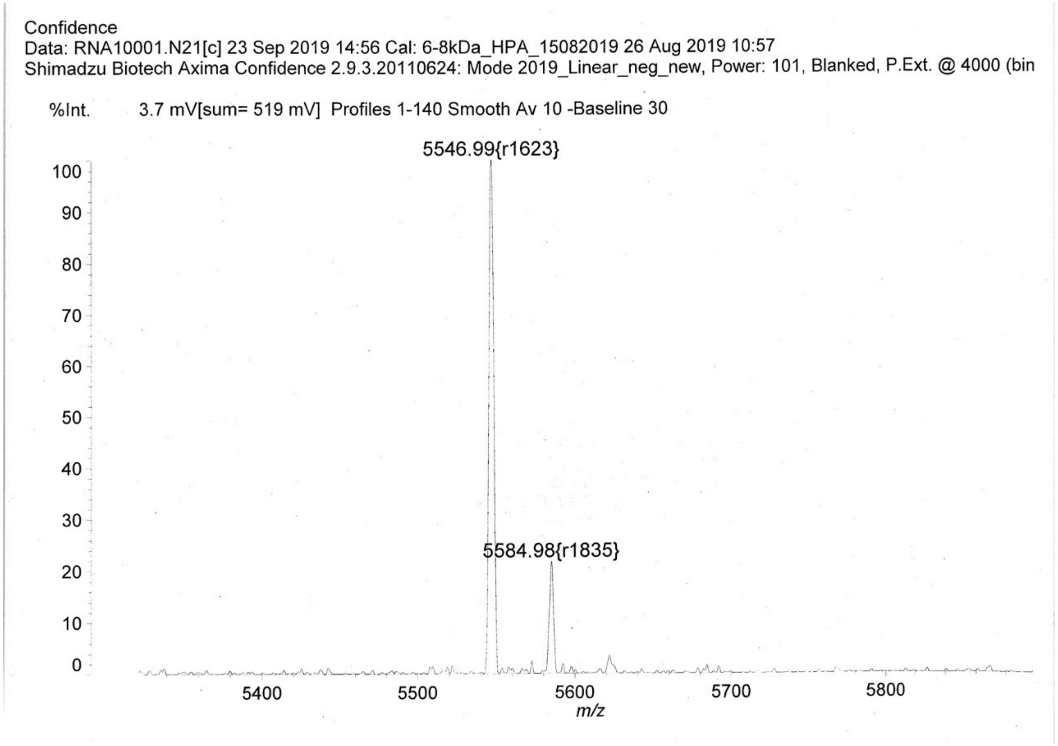

Figure S42. MS (MALDI-TOF) analysis of **RNA1**. Calculated mass [M^+]: 5544.6; m/z=5546.99 [M^+], 5584.98 [$M+K^+$].

biomolecules

MDPI

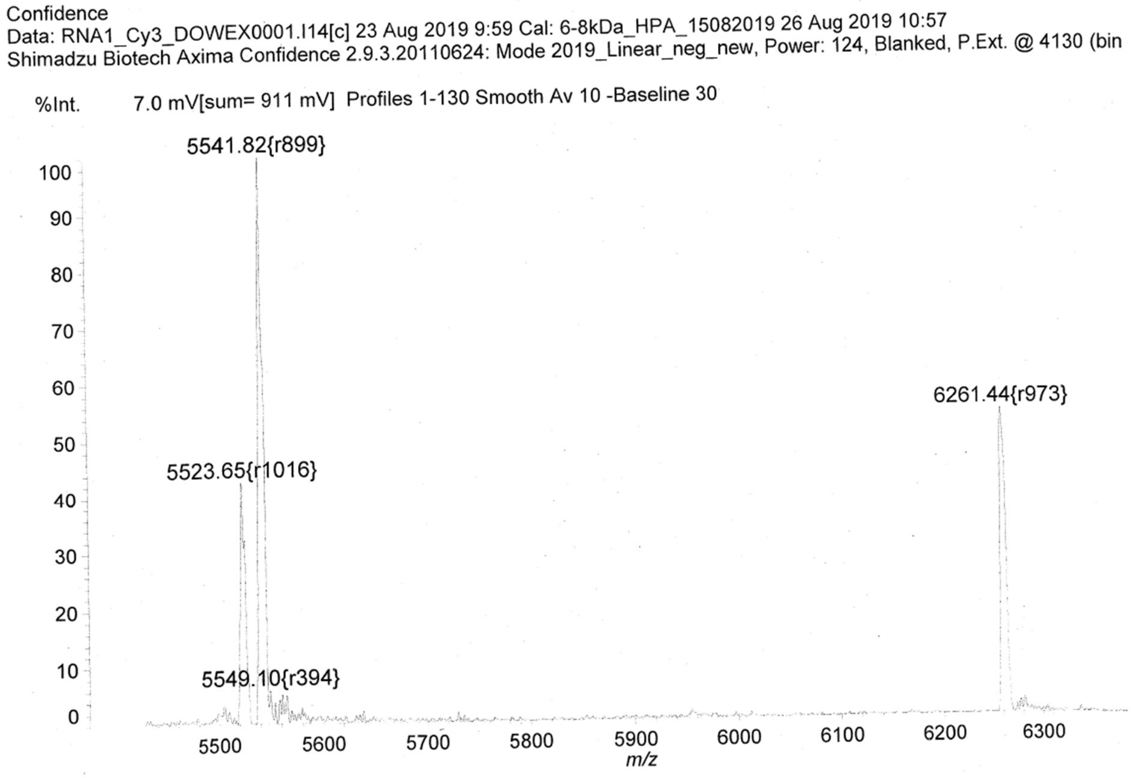

Figure S43. MS (MALDI-TOF) analysis of **RNA1**-Cy3 adduct. Calculated mass [M^+]: 6253.9; m/z=5523.65 [RNA1-N_2^+], 5541.82 [RNA1-N_2+H_2O^+], 6261.44 [M^+].

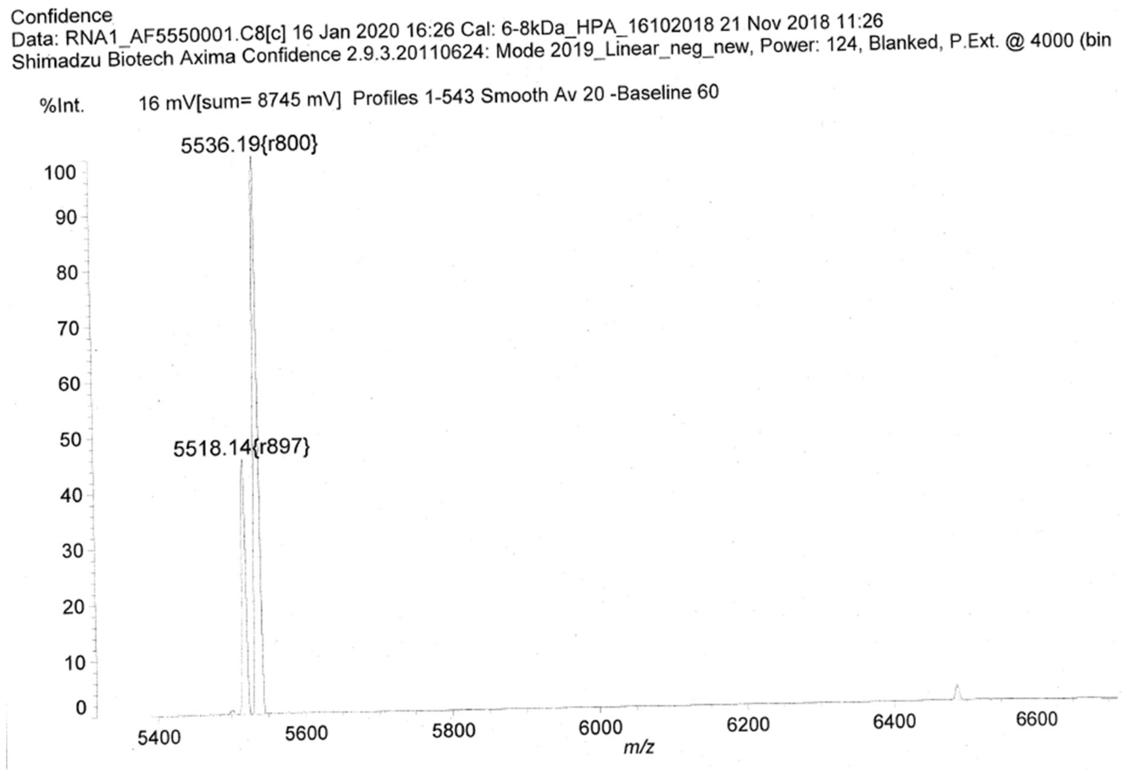

Figure S44. MS (MALDI-TOF) analysis of **RNA1**-AF555 adduct. Calculated mass: 6485.9 [M^+]; m/z=5518.14 [RNA1-N_2^+], 5536.19 [RNA1-N_2+H_2O^+], 6486.22 [M^+]. The molecular mass of AF555-maleimide was reported in literature and verified by MS (MALDI-TOF) analysis.[1]

biomolecules

MDPI

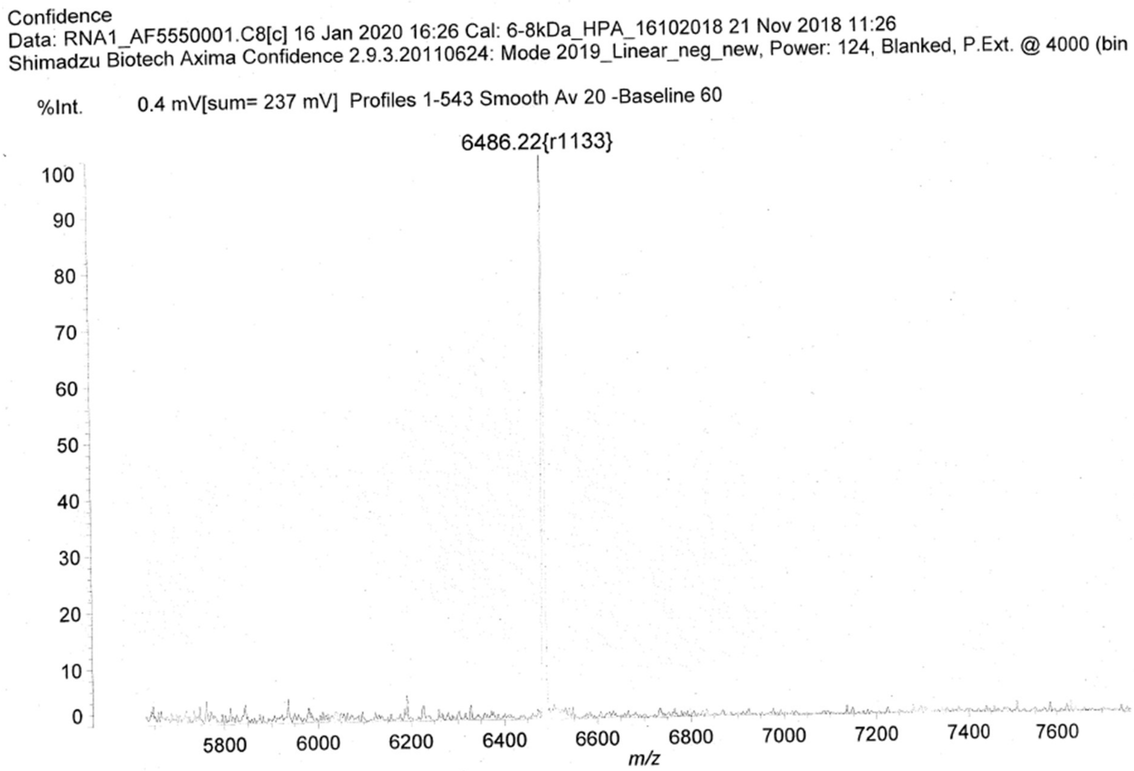

Figure S45. Zoomed area of MS (MALDI-TOF) analysis (Figure S44) of **RNA1**-AF555 adduct.

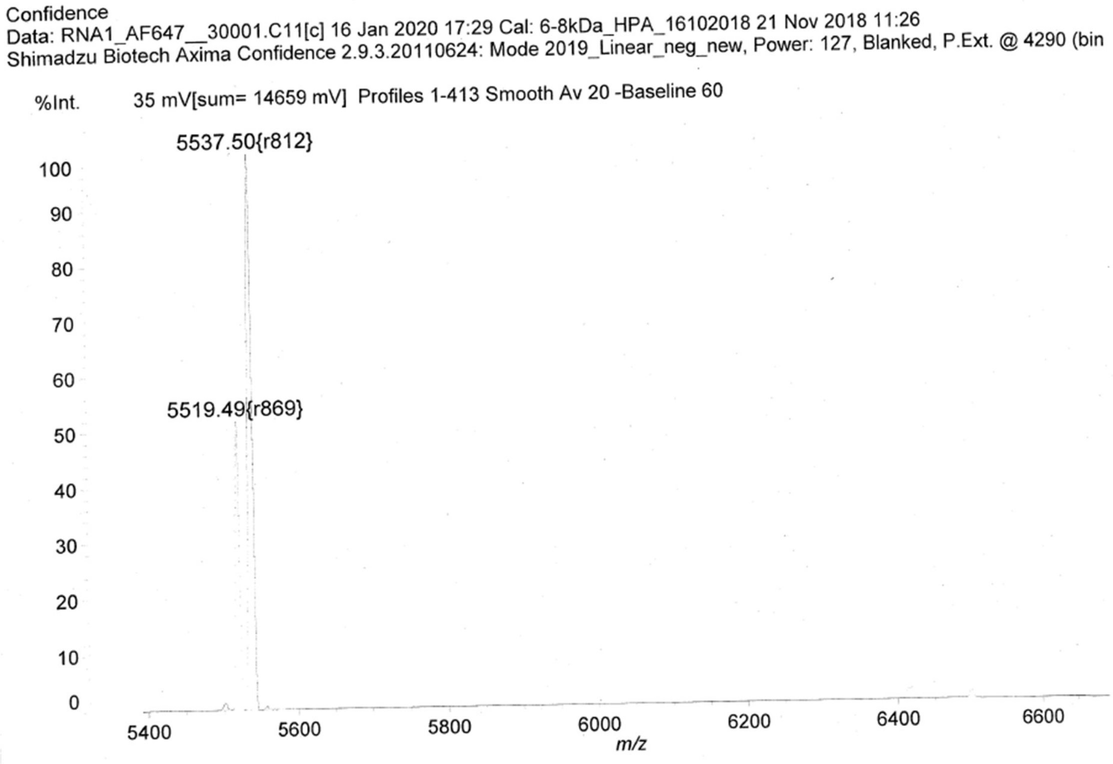

Figure S46. MS (MALDI-TOF) analysis of **RNA1**-AF647 adduct. Calculated Mass [M^+]: 6497.9; m/z=5519.49 [RNA1-N_2^+], 5537.50 [RNA1-N_2+$H_2$0$^+$], 6499.90 [M^+]. The molecular mass of AF647-maleimide was reported in literature and verified by MS (MALDI-TOF) analysis.[1]

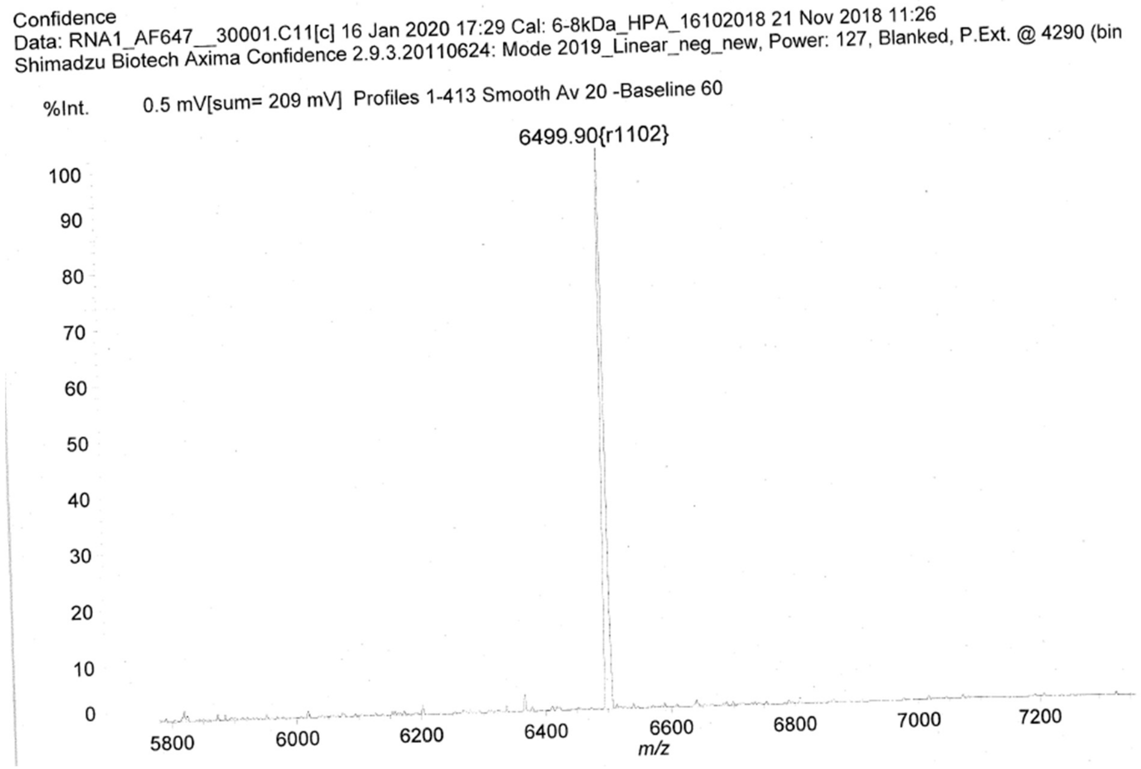

Figure S47. Zoomed area of MS (MALDI-TOF) analysis (Figure S46) of **RNA1**-AF647 adduct.

RNA2

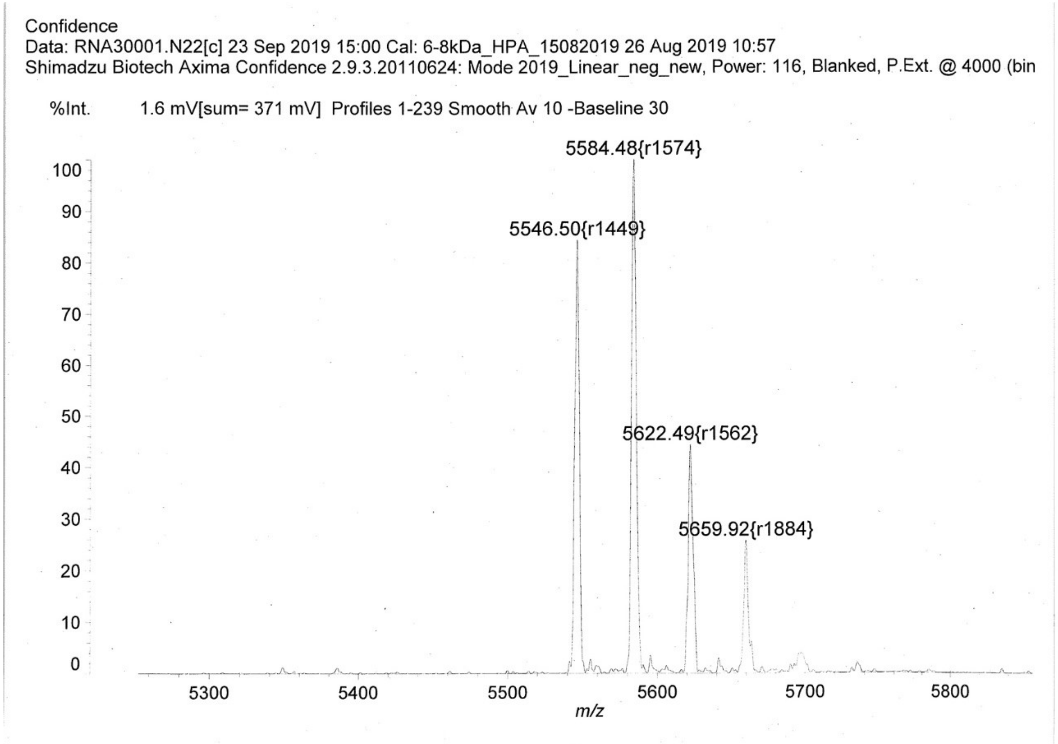

Figure S48. MS (MALDI-TOF) analysis of **RNA2**. Calculated mass [M$^+$]: 5544.6; m/z= 5546.50 [M$^+$], 5584.48 [M+K$^+$],5622.49 [M+2K$^+$], 5659.92 [M+3K$^+$].

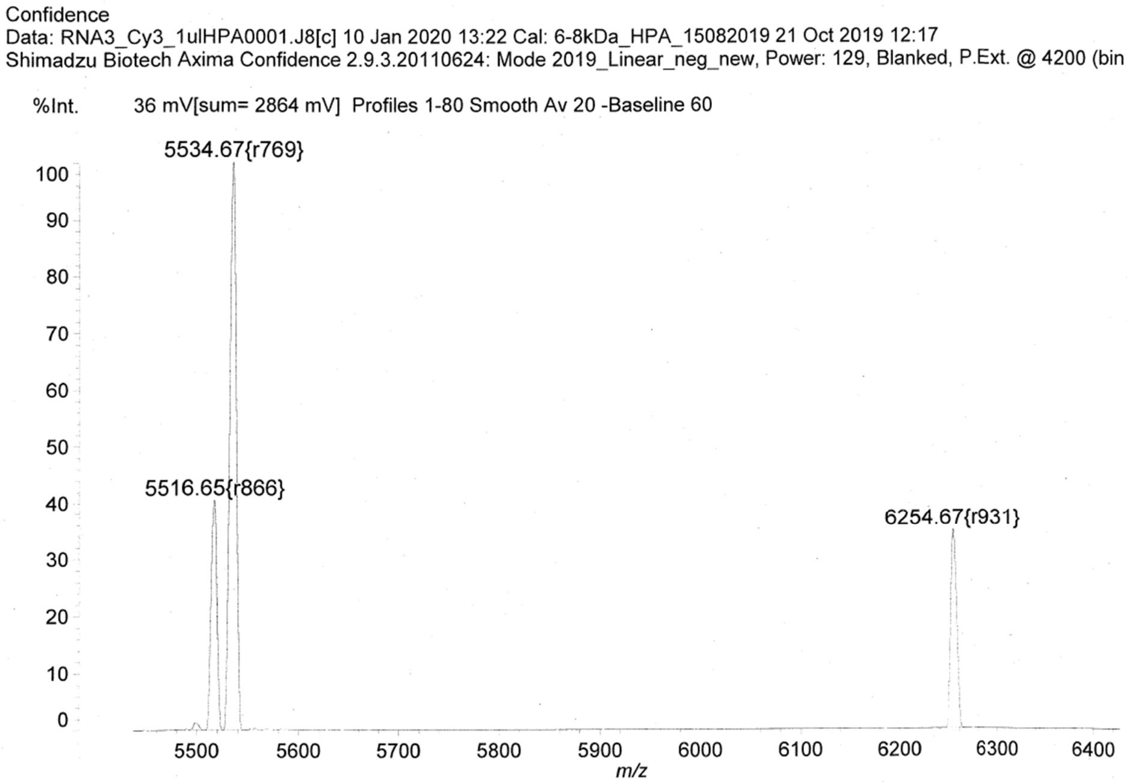

biomolecules

MDPI

Figure S49. MS (MALDI-TOF) analysis of **RNA2**-Cy3 adduct. Calculated mass [M^+]: 6253.9; m/z=5516.65 [RNA2-N_2^+], 5534.67 [RNA2-N_2+H_2O^+], 6254.67 [M^+].

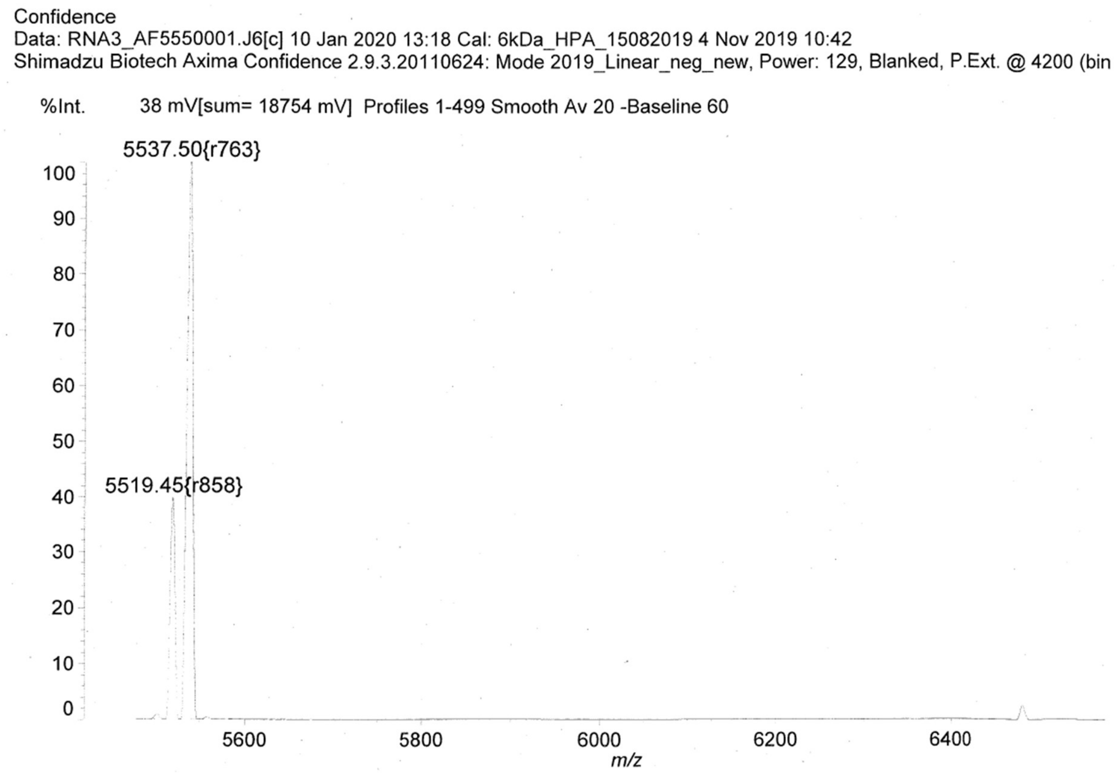

Figure S50. MS (MALDI-TOF) analysis of **RNA2**-AF555 adduct. Calculated Mass [M^+]: 6485.9; m/z=5519.45 [RNA2-N_2^+], 5537.50 [RNA2-N_2+H_2O^+], 6485.06 [M^+]. The molecular mass AF555-maleimide was reported in literature and verified by MS (MALDI-TOF) analysis.[1]

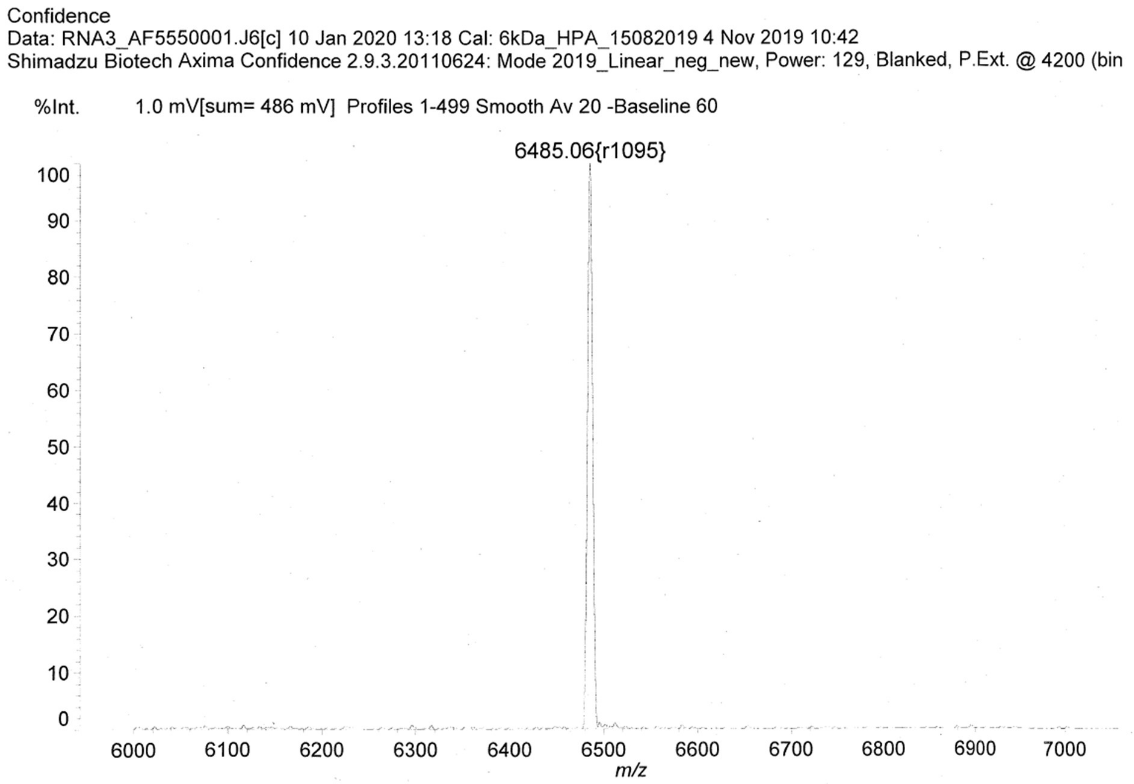

Figure S51. Zoomed area of MS (MALDI-TOF) analysis (Figure S50) of **RNA2**-AF555 adduct.

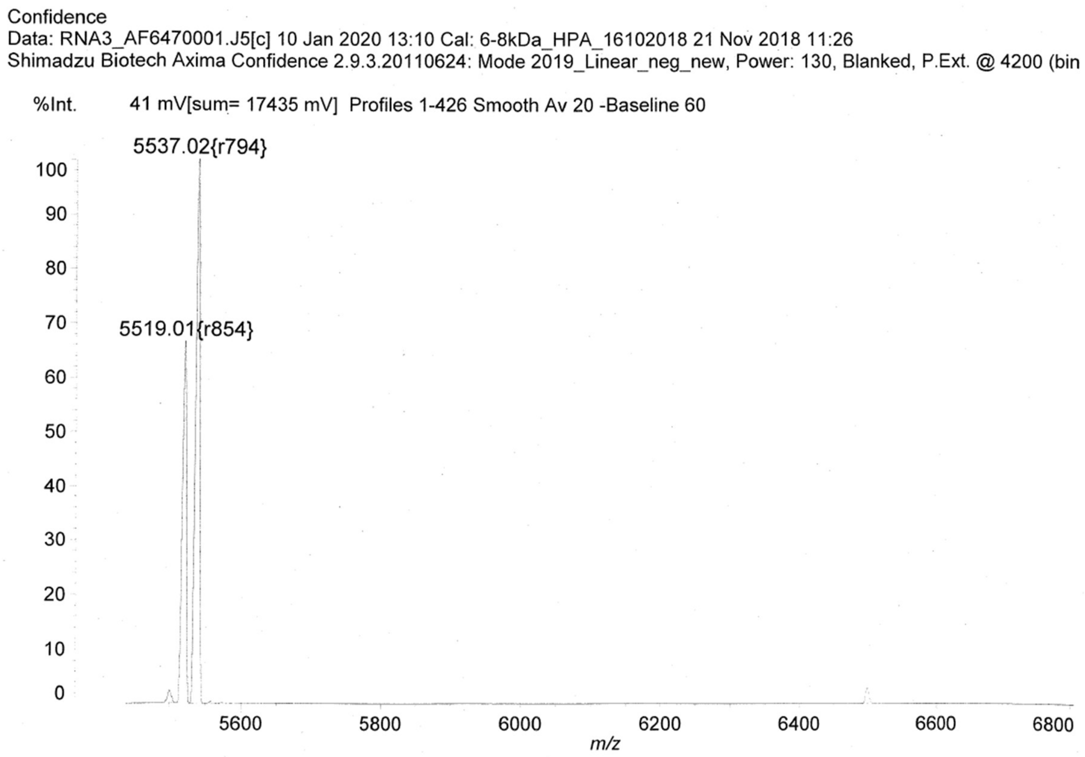

Figure S52. MS (MALDI-TOF) analysis of **RNA2**-AF647 adduct. Calculated mass [M^+]: 6497.9; m/z=5519.01 [RNA2-N_2^+], 5537.02 [RNA2-N_2+H_2O^+], 6499.74 [M^+]. The molecular mass AF647-maleimide was reported in literature and verified by MS (MALDI-TOF) analysis.[1]

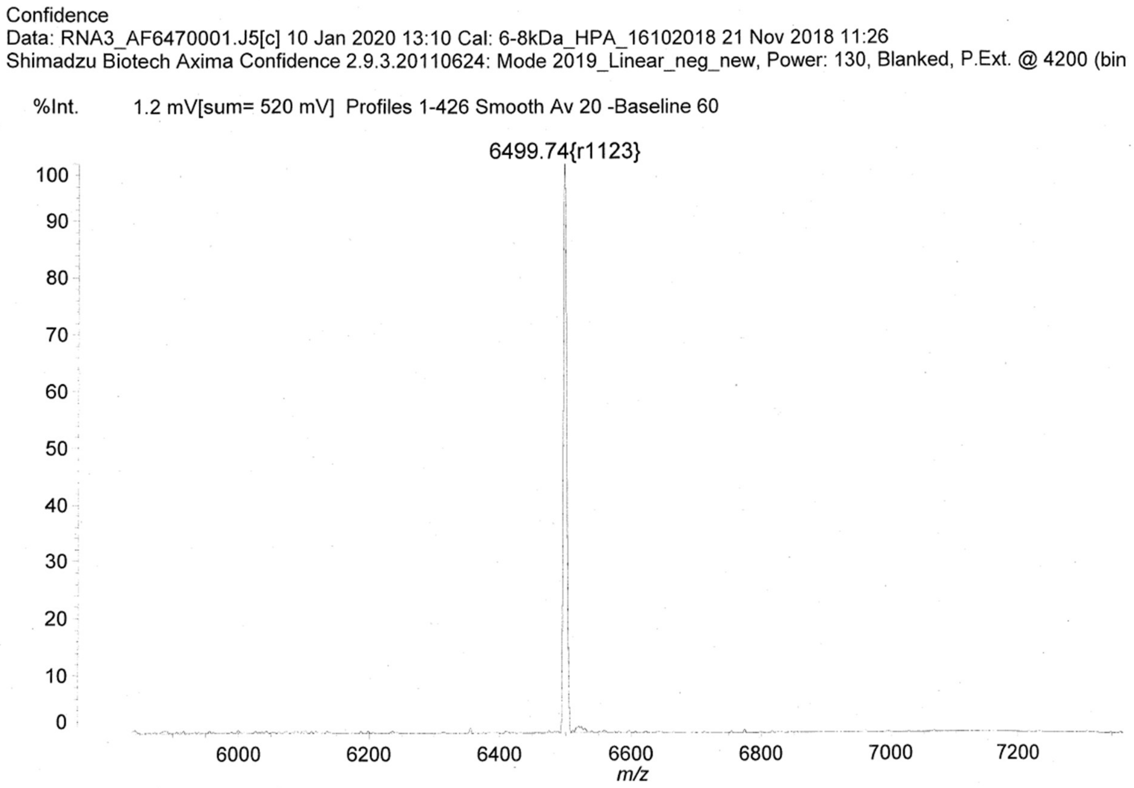

Figure S53. Zoomed area of MS (MALDI-TOF) analysis (Figure S53) of **RNA2**-AF647 adduct.

4. Determination of yields

A solution of RNA (2.5 µM) and of the dye (3.75 µM) in 10 mM Na-P_i buffer, 250 mM NaCl, pH 7, with a total volume of 500 µL was irradiated at 300 nm (LED) in a 10 mm quartz glass cuvette for 30 minutes. To remove the excess dye, the solution was purified *via* illustra™ NAP-5 columns (GE Healthcare) using the standard protocol. The eluted sample was lyophilized and redissolved in water (500 µL). The concentration was calculated spectroscopically by Lambert-Beer-Law using the extinction coefficient provided by the manufacturers of the clicked dye: ε_{548} (Cy3) = 162 000 L mol^{-1} cm^{-1} (Lumiprobe); ε_{555} (AF555) = 158 000 L mol^{-1} cm^{-1} (JenaBioscience); ε_{648} (AF647) = 270 000 L mol^{-1} cm^{-1} (JenaBioscience).

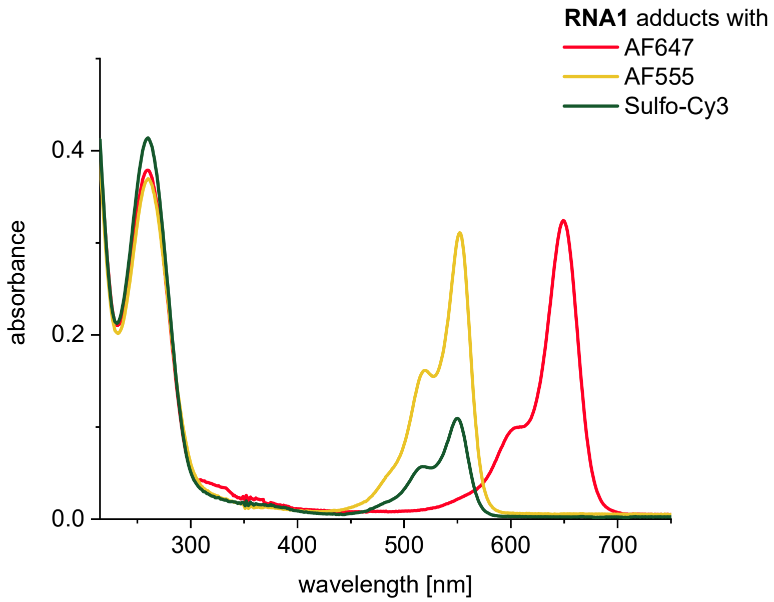

Figure S54. UV/vis absorbance of "photoclicked" **RNA1** dye adducts (reaction with 1.50 equiv. dye-maleimide) strands after purification. c_{AF647}=1.19 µM ≙ 48% yield, c_{AF555}=1.96 µM ≙ 78% yield, c_{Cy3}=0.67 µM ≙ 27% yield.

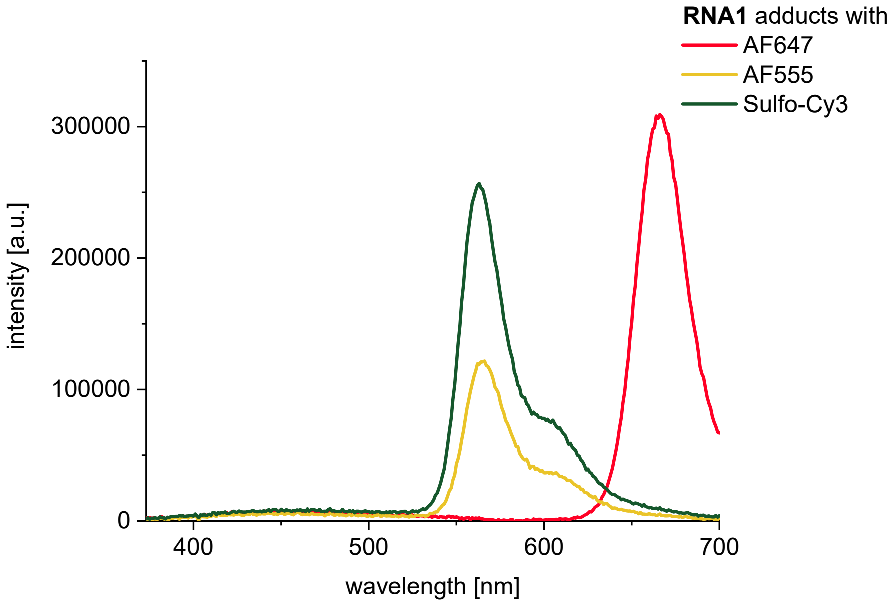

Figure S55. Fluorescence of "photoclicked" **RNA1** dye adducts (reaction with 1.50 equiv. dye-maleimide) after purification. c_{AF647}=1.19 µM, c_{AF555}=1.96 µM, c_{Cy3}=0.67 µM.

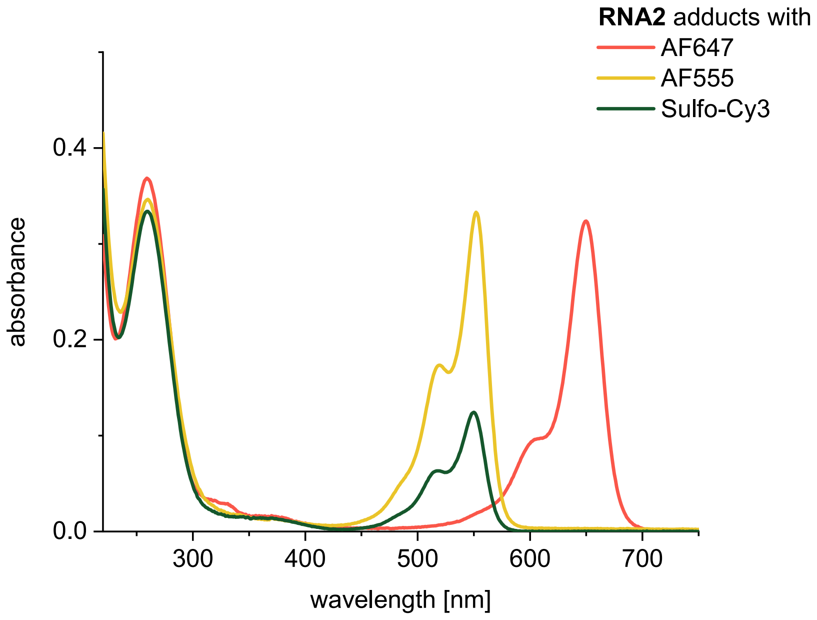

Figure S56. UV/vis absorbance of "photoclicked" **RNA2** dye adducts (reaction with 1.50 equiv. dye-maleimide) after purification. c_{AF647}=1.20 µM ≙ 48% yield, c_{AF555}=2.10 µM ≙ 84% yield, c_{Cy3}=0.77 µM ≙ 31% yield.

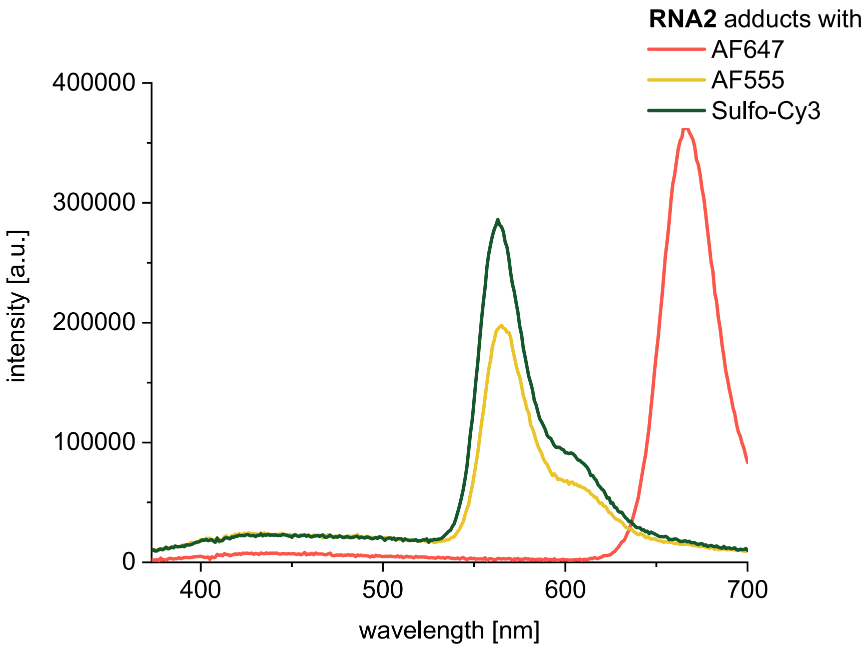

Figure S57. Fluorescence of "photoclicked" **RNA2** dye adducts (after reaction with 1.50 equiv. dye-maleimide) after purification. c_{AF647}=1.20 µM, c_{AF555}=2.10 µM, c_{Cy3}=0.77 µM.

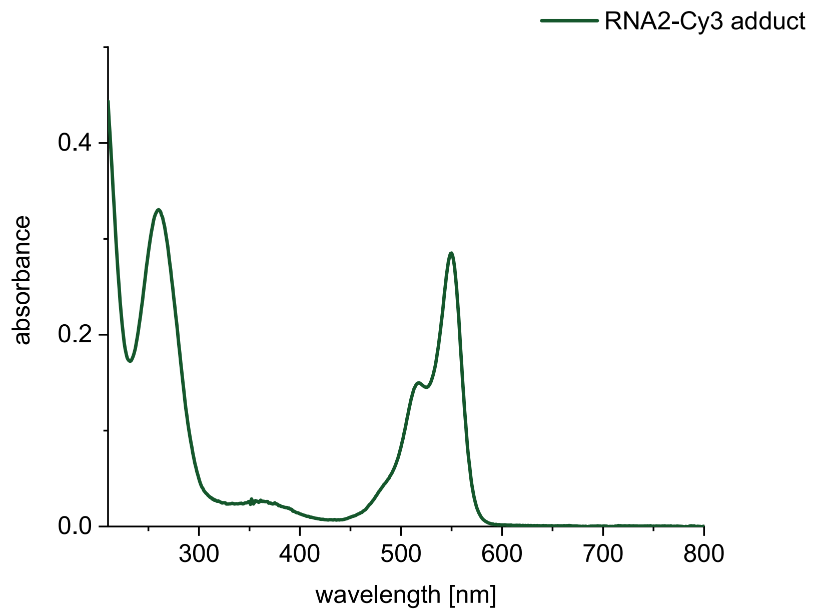

Figure S58. UV/vis absorbance of "photoclicked" **RNA1**-Cy3 adduct (reaction with 10.0 equiv. Cy3-maleimide) after purification. c_{Cy3}=1.76 µM ≙ 70% yield.

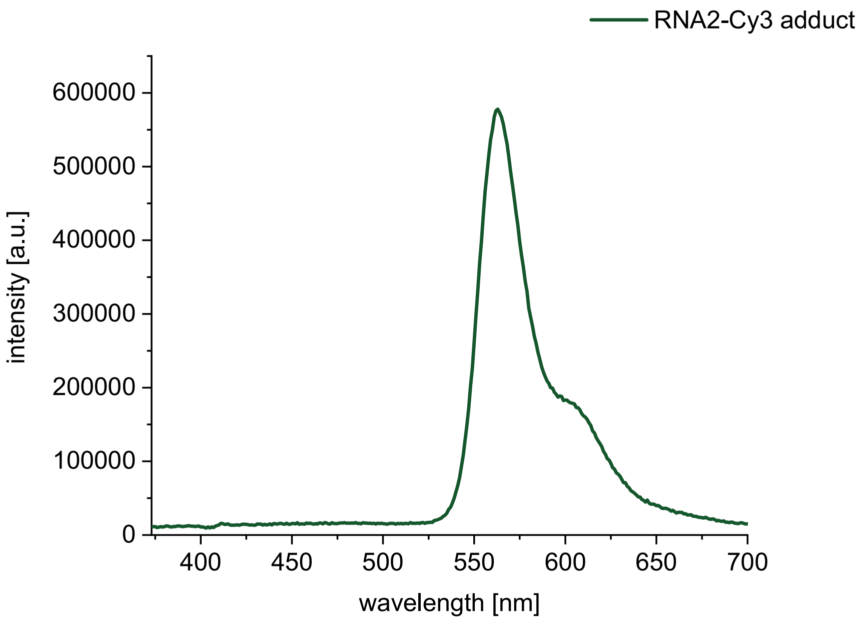

Figure S59. Fluorescence spectrum of "photoclicked" **RNA2**-Cy3 adduct (reaction with 10.0 equiv. Cy3-maleimide) after purification. c_{Cy3}=1.76 µM.

5. Calculation of extinction coefficients

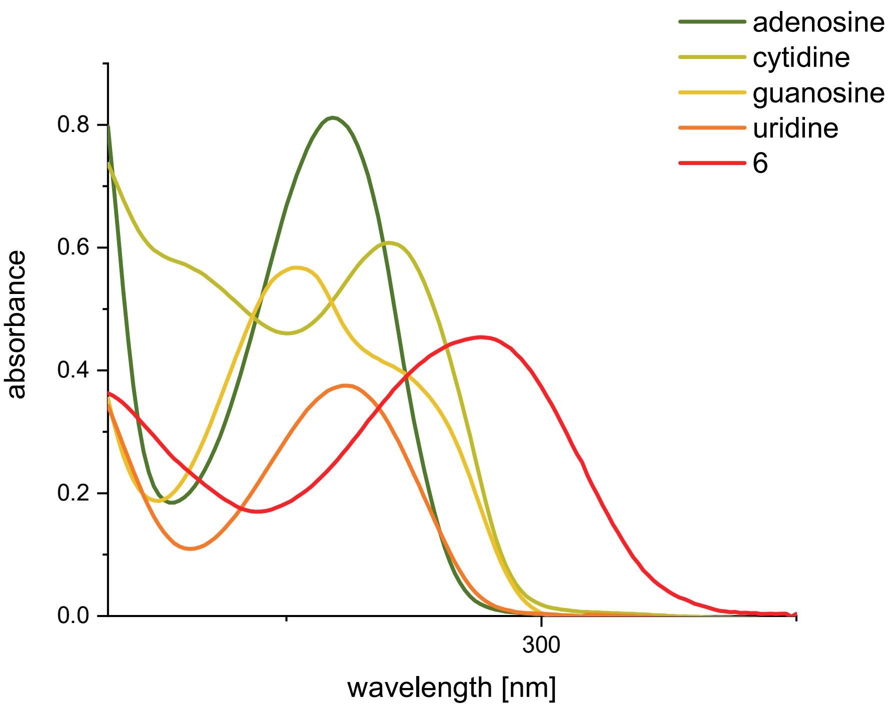

Figure S60. UV/vis absorbance of A, C, G, U and **6** in comparison.

The molar extinction coefficient ε_{300} were calculated for the natural nucleosides and the artificial building block **6** using the ε_{260} values and the recorded UV/vis absorbances (Figure S60) using the Lambert-Beer-Law.

Table S1. Molar extinction coefficients of the natural bases and the artificial nucleoside **6**.

nucleoside	ε_{260} [L mol^{-1} cm^{-1}]	concentration [µmol L^{-1}]	ε3$_{00}$ [L mol^{-1} cm^{-1}]
Adenosine	15,400	52.6	≈60
Cytidine	7,400	70.9	≈260
Guanosine	11 500	42.8	≈110
Uridine	8,700	42.9	≈90
**6**	13,800	18.4	20,300

6. References

1. Tridgett, M.; Moore-Kelly, C.; Duprey, J.-L. H. A.; Iturbe, L. O.; Tsang, Chi W.; Little, H. A.; Sandhu, S. K.; Hicks, M. R.; Dafforn, T. R.; Rodger, A., Linear dichroism of visible-region chromophores using M13 bacteriophage as an alignment scaffold. *RSC Adv.* **2018,** *8* (52), 29535-29543.
